# Assessing protein model quality based on deep graph coupled networks using protein language model

**DOI:** 10.1093/bib/bbad420

**Published:** 2023-11-28

**Authors:** Dong Liu, Biao Zhang, Jun Liu, Hui Li, Le Song, Guijun Zhang

**Affiliations:** College of Information Engineering, Zhejiang University of Technology; College of Information Engineering, Zhejiang University of Technology; College of Information Engineering, Zhejiang University of Technology; researcher of AI in the BioMap; Chief Scientist of AI in the BioMap & MBZUAI; College of Information Engineering, Zhejiang University of Technology

**Keywords:** protein model evaluation, protein language model, graph neural network, multimer model evaluation

## Abstract

Model quality evaluation is a crucial part of protein structural biology. How to distinguish high-quality models from low-quality models, and to assess which high-quality models have relatively incorrect regions for improvement, are remain a challenge. More importantly, the quality assessment of multimer models is a hot topic for structure prediction. In this study, we propose GraphCPLMQA, a novel approach for evaluating residue-level model quality that combines graph coupled networks and embeddings from protein language models. The GraphCPLMQA consists of a graph encoding module and a transform-based convolutional decoding module. In encoding module, the underlying relational representations of sequence and high-dimensional geometry structure are extracted by protein language models with Evolutionary Scale Modeling. In decoding module, the mapping connection between structure and quality is inferred by the representations and low-dimensional features. Specifically, the triangular location and residue level contact order features are designed to enhance the association between the local structure and the overall topology. Experimental results demonstrate that GraphCPLMQA using single-sequence embedding achieves the best performance compared with the CASP15 residue-level interface evaluation methods among 9108 models in the local residue interface test set of CASP15 multimers. In CAMEO blind test (20 May 2022 to 13 August 2022), GraphCPLMQA ranked first compared with other servers (https://www.cameo3d.org/quality-estimation). GraphCPLMQA also outperforms state-of-the-art methods on 19, 035 models in CASP13 and CASP14 monomer test set.

## INTRODUCTION

Protein structure prediction plays an important role in biological research. In recent years, the development of deep learning has greatly advanced the transformation and progress of protein structure prediction. Many high-accuracy deep learning structure prediction methods have been developed, such as AlphaFold2 [1], RoseTTAFold [2], ESMFold [3], RGN2 [4] and PAthreader [5]. More impressively, the collaboration between the European Molecular Biology Laboratory and DeepMind has predicted structures for over 200 million proteins and made them freely available at the AlphaFold Protein Structure Database [6]. While AlphaFold2’s internal confidence estimate is important, it may not be the only metric for assessing the quality of a predictive model. With the breakthrough of structure prediction, the reliability and usability of models are crucial parts, which are directly related to the efficiency of target discovery and drug design. Model quality assessment is important for structure prediction. Needless to say more, model quality assessment can further improve the accuracy of protein structure [7], and can also screen out the relatively best structure from multiple candidate models, which is critical for experimental scientists to analyze and verify.

Since CASP7, many methods for assessing the quality of protein models have been developed [8–10]. In particular, single-model evaluation methods have received increasing attention and research, because they require only one model as input and show similar or better performance than consensus methods [11, 12]. Features and networks are important for single-model quality assessment using deep learning. Features can explicitly describe the properties of proteins that include protein structural and nonstructural features. For structural feature representation, some methods calculate inter-residue distances from atomic coordinates of protein models, and transform distances through spatial mapping to reflect the local structure and overall topology [13, 14]. However, these methods only describe simple low-order distance relationships of protein geometric models and may ignore infrastructural connections in high-dimensional spaces. For nonstructural feature representations, the Rosetta energy [15, 16] and statistical potential of the model represent the physicochemical information of the protein, such as ProQ3 [17] and VoroMQA [18]. Particularly, sequence information implies the evolutionary relationship of proteins, which can improve the accuracy of model quality, such as ProQ4 [19] and DeepAccNet-MSA [7]. These methods just use sequence alignment information, it is more important to establish sequence–structure relationship. In addition, for our in-house model quality assessment method, DeepUMQA [20] designed the residue-level USR [21] feature to characterize the topological relationship between the residuals and the overall structure. The improved version DeepUMQA2 [22] significantly improves the accuracy of model quality assessment by introducing co-evolution and template information, supplemented by an improved attention mechanism network framework. However, there is still space for improvement in network architecture.

Deep learning networks can capture potential connections within proteins. Various neural networks that contain convolution, LSTM and graph networks, are used in model quality assessment methods, as ProQ3D [23], AngularQA [24] and GraphQA [25]. These methods use specific neural network architectures and build only one learning module. The learning mode of the network may be single, and the connection between the network architectures is not well utilized. Building blocks for specialized learning may help improve prediction accuracy. DeepAccNet utilizes 3D convolutional networks to obtain local atomic structure information, and then uses 2D convolutions to predict model quality. In addition, AlphaFold2 utilizes evolutionary blocks to encode sequence information and predict atomic coordinates and structural quality in structural modules. Therefore, in model quality assessment, the network forms an encoder-decoder architecture, which can establish the connection among sequence, structure and quality to help improve the accuracy of model quality. The previous research studies show that structure, sequence, physicochemical information and deep learning network architecture are crucial for model quality assessment.

Protein language models are widely used in protein modeling and design tasks, which are trained unsupervised on protein databases to obtain embedding representations. In protein modeling tasks, sequence embeddings from protein language models are used to infer structural information, such as IgFold [26], ESMFold [3] and RGN2 [4]. In sequence design tasks, structural embeddings from backbone atomic coordinates are used to predict protein sequences by networks, as ESM-IF1 [27]. These methods show that protein language models establish an abundant connection between sequence and structure, which open the possibility of using language models in model quality assessment.

In this work, we propose GraphCPLMQA based on a deep graph coupled neural network framework using protein language models. Embeddings representations are generated by the protein language model ESM, which reflect sequence and structural properties. The embeddings that supplemented by structural features are input into a deep graph coupled network. The network consists of two parts: (i) the graph encoding network learns the latent connection between sequence and structure. (ii) the transform-based convolutional decoding network obtains the mapping relationship between structure and quality to evaluate protein models. The results show that representations from language models and graph-coupled neural networks can learn the implicit relationship among the sequence, structure and quality, which further improve the accuracy of model quality.

## MATERIALS AND METHODS

### Overview

**Figure 1:**
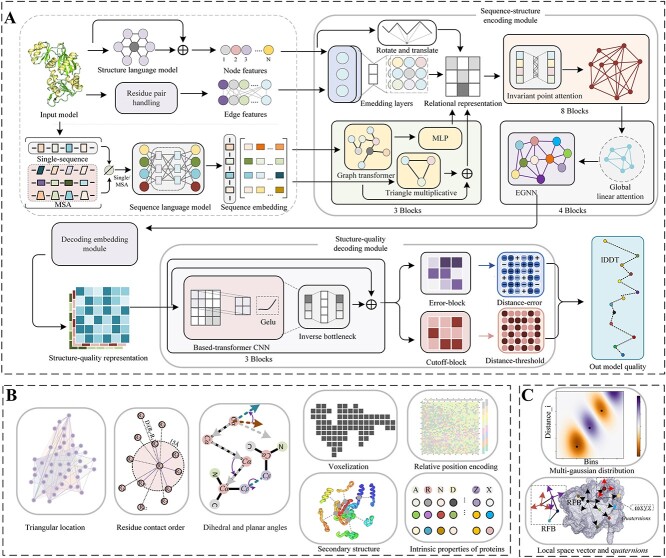
(**A**) The workflow of GraphCPLMQA. We extract the features in (**B**) and (**C**) along with the embedding representation from the protein structure where Single/MSA means that the input is single-sequence or MSA information corresponding to getting a single-sequence embedding or MSA embedding. In the sequence-structure encoding module, we generate the relational representation of sequence and structure, which inputs to the structure-quality decoding module. Finally, the graph coupled network outputs the results of evaluating the model.

In this section, we described the GraphCPLMQA method in three parts, including training datasets, input features for proteins and network architecture. In addition, we provide two versions according to different sequence embedding types, namely the full version of GraphCPLMQA (GraphCPLMQA-MSA) and the single sequence version of GraphCPLMQA (GraphCPLMQA-single). The pipeline is shown in Figure 1.

### Train dataset

The training dataset of GraphCPLMQA was constructed from the Protein Data Bank (PDB). A total of 15, 054 proteins were selected from the PDB (19 November 2021) based on the following criteria: (i) minimum resolution <=2.5 Å, (ii) protein length within 50 ~ 400 residues, (iii) sequence similarity to any protein in the dataset <35%.

For each protein of the training set, three different approaches were used to generate decoys (structure models): structural dihedral adjustment, template modeling and deep learning-guided conformational changes (Supplement Figure S1). For structural dihedral adjustment, dihedral angles were fine-tuned on experimental structure (proteins from PDB). Each adjustment was followed by a fast relaxation process. For template modeling, RosettaCM [28] and I-TASSER-MTD [29] were used to generate diverse structural models by utilize template structures with different accuracy and fragment libraries. For deep learning-guided conformational changes, our in-house method RocketX [30] was used to generate models by setting different geometric constraint weights. In addition, the constrained conformations were refined to produce diverse models. Finally, after filtering for similar structures, a total of 1,378, 676 protein models were obtained and utilized for training graph coupled network.

### Protein language embedding features

Most current model quality assessment methods use one-hot encoding for sequence embedding. However, there is sometimes a problem with only using one-hot encoding. One-hot encoding cannot effectively represent the similarity or difference between amino acids, as it may fail to capture the underlying relationships in protein sequences. In addition, mapping sequences to structures using one-hot encoding may burden model quality assessment networks focused on structure learning. To characterize the structural information of protein models, some methods describe the relative positions of residues in different coordinate systems, such as 3DCNN, Ornate, DeepAccNet and DeepUMQA. However, these methods do not consider the implicit connections of residues in higher-dimensional spaces.

In this work, embeddings of protein sequences and geometric structures from ESM were employed to capture sequence–structure relationships. We have devised two distinct versions of the methods to assess protein model quality, namely the GraphCPLMQA method, which utilizes the MSA language model, and the GraphCPLMQA-Single, which employs a single sequence language model. For GraphCPLMQA-Single, the residue-level sequence embedding (1280-dim) of the query sequence is generated by ESM2 [3] at the last layer of the network. For GraphCPLMQA, the MSA of the input structure model is first produced through HHbits [31] searching against UniRef30 [32] and BFD [33], and then the searched MSA is fed into the ESM-MSA-1b [34] language model to derive residue-level sequence embeddings (768-dim) and row-attention embeddings between residues (144-dim) from the last layer (12th) of the network. Both versions of GraphCPLMQA utilize the ESM-IF1 [27] to generate the structural embedding (512-dim) of the input backbone atomic coordinates. It should be noted that the input sequence length of ESM is limited to 1022, and the sequence length exceeding this limit will be processed. If the length of the input sequence is >1022 but <2044, the sequence is truncated into two sequences from the middle, and they are input into the ESM language model to obtain the corresponding embedding representation. These representations are then sequentially reassembled as feature inputs to the model evaluation network. For information on protein language models, see Supplementary Table S1.

### Triangular location and residue level contact order

To describe the protein structure, the triangular location feature was designed, which is inspired by the residue-level USR from DeepUMQA [20]. The feature characterizes the orientation and distance of the local structure in the overall topology. To construct the triangular location feature, the farthest point ${P}_2^i$ was identified for the C_α_ coordinate of residue ${P}_1^i$ in the protein structure, and ${P}_2^i$ was taken as the center to find the farthest point ${P}_3^i$ (excluding ${P}_1^i$). These three points formed a triangle ${S}_i$, with *N* (number of residues) small triangles outlining the fundamental shape of the protein structure. In each triangle ${S}_i$, the side lengths were ${D}_i^{1,2}$, ${D}_i^{1,3}$, ${D}_i^{2,3}$, and the average distance from all residues to these three points were calculated as ${D}_i^{ave1}$, ${D}_i^{ave2}$, ${D}_i^{ave3}$. Finally, a local coordinate system ${\Gamma}_i$ was constructed with ${P}_1^i$ to characterize the position of the triangle in space. The local coordinate system described the orientation of the local structure in the overall topology. The calculation process is as follows:


(1) \begin{equation*} {e}_x=\frac{P_3^i-{P}_1^i\ }{\left\Vert{P}_3^i-{P}_1^i\right\Vert } \end{equation*}

(2) \begin{equation*} V={P}_2^i-{P}_1^i \end{equation*}

(3) \begin{equation*} {e}_y=\frac{V-{V}_{ex}}{\left\Vert V-{V}_{ex}\right\Vert } \end{equation*}

(4) \begin{equation*} \Theta = Euler\left({e}_x,{e}_y,{e}_x\times{e}_y\right) \end{equation*}

where ${V}_{ex}$ denotes the projection length of $V$ on ${e}_x$ multiplied by ${e}_x$ to obtain a projection vector. In the above equation, $Euler$ represents the mapping function from the local coordinate system to the Euler angles.

Contact order [35] is used to describe the overall topology complexity. We further extend to residue-level features to describe the complexity of local structures and between local structures. The calculation process is as follows:


(5) \begin{equation*} {O}_i=\sum_{i\ne j}\frac{\left|i-j\right|}{R_iN} \end{equation*}

(6) \begin{equation*} {O}_{ij}=\frac{\left|i-j\right|}{d_{ij}} \end{equation*}

where $i$, $j$ are indeces of residue; ${R}_i$ is the number of adjacent residues within 15 Å for residue $i$; ${d}_{ij}$ is the distance between residue $i$ and residue $j$; $N$ is protein length.

### Protein node features and edge features

The positional order and properties of amino acids are critical to protein structure. The relative position encoding method in Transformer [36] was employed to encode the sequence order. Each residue *i* finds the closest *K* residues in the space and records their relative indexes, which are converted into node features through the position encoding formula. See Supplementary Text S1 for specific details. The properties of amino acids are represented by Meiler [37] and Blosum62 [38]. To characterize the information of the secondary structure, DSSP [39] was used. The voxelization [14] of protein structures with rotation-translational invariance further complements the overall topological information, see Supplementary Text S2 for specific details. To capture the spatial arrangement of residues within the protein structure, vectors between backbone atoms are used to represent dihedral and plane angles. Local vector ${s}_{ij}^{\Gamma}$ is used to describe the relative positional relationship of residues, and the rotation transformation ${Q}_{ij}$ represents the relationship between each local spatial structure (see Supplementary Text S3 for details). To map the distances from the main chain atoms to a high-dimensional space, different interval Gaussian functions are employed to disperse the distances. In addition, distance map features are computed between the ${C}_{\beta }$ atoms and the tip atoms [7], which complement the edge information of the graph network. The inter-residue Rosetta energy terms are used to represent the physicochemical information of the protein. The detailed dimension information of all features is in the Supplementary Table S2.

### Sequence-structure encoding module

In the encoding module, a protein graph is typically represented as $\mathcal{G}=\left(\mathcal{V},\mathcal{E},\mathcal{X}\right)$. In the $\mathcal{G}$ protein graph, $\mathcal{V}=\left\{{\upsilon}_1,{\upsilon}_2,\dots, {\upsilon}_N\right\}$ is the set of residues, $\mathcal{E}={\left\{{\varepsilon}_{ij}\right\}}_{i\ne j}$ is the set of edges between residues, where each ${\varepsilon}_{ij}\in{\mathbb{R}}^{d_e}$ is the feature vector between residue $i$ and residue $j$, and $\mathcal{X}=\left\{{x}_i\in{\mathbb{R}}^{3\times 5}\right\}$ represents the coordinates $C,O,N,{C}_{\alpha },{C}_{\beta }$ coordinates of the backbone atoms for residue $i$.

In the triangle graph transformer [40], the residual embedding ${v}_i^{plm}$ and attention between residues ${e}_i^{plm}$ from the protein language model are input the module. It allows for a deeper exploration of spatial geometric information and the potential relationship between sequence and structure. For the graph transformer layer, each residue $i$ attends to all other residues $j$ using multi-head attention as follows [40]:


(7) \begin{equation*} {q}_i,{k}_i={\varphi}_i\left({v}_i,{v}_j\right), \end{equation*}

(8) \begin{equation*} {m}_{ij}={\psi}_{ij}\left({\varepsilon}_{ij}\right), \end{equation*}

(9) \begin{equation*} {C}_{ij}=\frac{e^{\left\langle{q}_i,{k}_j+{m}_{ij}\right\rangle }}{\sum_{t=1}^N{e}^{\left\langle{q}_i,{k}_{jt}+{m}_{it}\right\rangle }} \end{equation*}

Where ${\psi}_{ij},{\varphi}_i$ represent trainable linear functions, which map from ${v}_i,{v}_j,{\varepsilon}_{ij}$ to ${q}_i,{k}_i,{m}_{ij}$, respectively. $\left\langle X,Y\right\rangle =\frac{X^TY}{\sqrt{A_d}}$ is used to scale the dot product attention operation between two matrices, ${A}_d$ is the dimension of attention. The attention for residue $i$ is computed with all residues $j$ as follows, and weighs the updated sequence embedding ${\overline{h}}_i$ with the original sequence embedding ${h}_i$ by gating follows [40]:


(10) \begin{equation*} {\overline{h}}_i={\parallel}_k\sum_{j=1}^N{C}_{ij}\left({W}_j{h}_j+{m}_{ij}\right), \end{equation*}

(11) \begin{equation*} {C}^i= sigmoid\left({\Phi}_s\left({h}_i-{h}_j,{h}_i,{h}_j\right)\right), \end{equation*}

(12) \begin{equation*} {h}_i^{update}={C}_1{h}_i+{C}_2{\overline{h}}_i, \end{equation*}

Where ${W}_j\in{\mathbb{R}}^{d_n\times{d}_m}$ is attention head learning matrix; ${\Phi}_s$ is trainable linear function ${\mathbb{R}}^{3{d}_n\times 1}$. In the above, $\parallel$ denotes splicing operation of multiple heads; $k$ is number of heads; ${C}_1,{C}_2$ are ${C}^i$ and $1-{C}^i$.

In invariant point attention [1], outputs of the triangle graph transformer are combined with the node and edge features of the model structure itself and input into the network to obtain geometric space constraints (rotations and translations) that are strongly associated with the sequence information. For the construction of the network layer, please refer to AlphaFold2’s invariant point attention mechanism.

To further fine-tune the node information, an Equivalent Graph Neural Network (EGNN) [41] is utilized. In the EGNN architecture, each node i searches the K nearest residual nodes in the Euclidean space to form a new graph $\hat{\mathcal{G}}=\left(\hat{\mathcal{V}},\hat{\mathcal{E}},\hat{\mathcal{X}}\right)$. For the new graph $\hat{\mathcal{G}}$, the output of the invariant point attention is further updated through the global linear attention layer and the graph equivariant layer as follows:


(13) \begin{equation*} {\hat{m}}_{ij}={\hat{\phi}}_m\left({\hat{\nu}}_i,{\hat{\nu}}_j, Fourier\left\Vert{\hat{x}}_i-{\hat{x}}_j\right\Vert, {\hat{\varepsilon}}_{ij}\right) \end{equation*}

(14) \begin{equation*} {\hat{x}}_i^{update}={\hat{x}}_i+\beta \sum_{i\ne j}\left({\hat{x}}_i-{\hat{x}}_j\right)\hat{W}{\hat{m}}_{ij} \end{equation*}

(15) \begin{equation*} {\hat{v}}_i^{update}={\hat{\phi}}_v\left(\sum_{i\ne j}{\hat{m}}_{ij},{\hat{\nu}}_i\right) \end{equation*}

Where ${\hat{\phi}}_m,{\hat{\phi}}_v$ are graph network trainable linear layers; $\hat{W}$ is learnable matrix; $Fourier\left\Vert{\hat{x}}_i-{\hat{x}}_j\right\Vert$ is fourier transform of the distance between node coordinates ${\hat{x}}_i$; $\beta$ is ${\left(N-1\right)}^{-1}$. The proof of equivariance is provided in Supplementary Text S4.

### Structure-quality decoding module

In the decoding embedding module, we extract the node representation ${\hat{\mathrm{\nu}}}_i$ and the edge representation ${\hat{\mathrm{\varepsilon}}}_{ij}$ from the output of the encoding module. Moreover, the representations and structural features are used by a new network function ${W}^{\ast }$ to generate new nodes ${v}_i^{\ast }$ and edges ${\varepsilon}_{ij}^{\ast }$ where the function utilizes the new parameters. These all features are combined to generate a structure-quality representation as follows:


(16) \begin{equation*} {m}_{ij}^{cat}= title\left({\hat{v}}_i\oplus{v}_i^{\ast}\right)\oplus{\hat{\mathrm{\varepsilon}}}_{ij}\oplus{\varepsilon}_{ij}^{\ast } \end{equation*}

where $\oplus$ denotes the concatenation of feature vectors; $title$ indicates horizontal striping of node features into edge features.

In the structure-quality decoding module, a residual network based on a transform strategy is employed, which consists of main residual blocks and branch residual blocks (Error-Block and Cutoff-Block). Each residual block comprises three 2-dimensional convolutional layers with different expansion rate coefficients and a normalization operation. We take the GELU [42] activation function and inverted bottleneck method, which is one important design in every transformer block [43]. Moreover, the convolutional network layer is added in the residual block of the branch to improve the prediction of distance error and threshold, as follows:


(17) \begin{equation*} {M}_{ij}^{update}={\left[{Conv}_r\left( GELU\left( Norm\left({m}_{ij}^{cat}\right)\right)\right)\right]}_{IB} \end{equation*}

where ${Conv}_r$ are 2-dimensional convolutional networks with different dilation coefficients $r={p}^2\left(p:1,\dots, 4\right)$; $IB$ is the operation of inverted bottleneck.

The distance-error ${M}_{ij}^e$ and the distance-threshold ${M}_{ij}^t$ are obtained from the base-transformer residual network. ${M}_{ij}^e$ is the predicted distance error between the real structure and the model structure, and the distance threshold ${M}_{ij}^t$ is the distance value within 15 Å where the threshold range is from lDDT [44]. Finally, we calculate the local quality score as follows:


(18) \begin{equation*} {M}_{ij}={M}_{ij}^e\odot{M}_{ij}^t \end{equation*}

(19) \begin{equation*} {PreLDDT}_i=\frac{\sum_j\sum_{s\in T}{\left({M}_{ij}\right)}_s}{\sum_j{M}_{ij}^t} \end{equation*}

Where $\odot$ denote distance error dot product distance thresholds to get the error within the threshold $s$ in 0.5, 1, 2, 4.

### Training procedure

The graph-coupled network model is trained using a combination of model quality and geometric constraint loss terms. To improve the efficacy of the sequence-structure encoding module, we use actual geometric structure information (real residue coordinates and distances) to constrain the encoding output (predicted residue coordinates and distances) by the mean square error between atomic coordinates and the L1 loss function. This approach helps in decoding the underlying information of structure and quality. In the decoding module, we compute the cross-entropy loss for the distance error and threshold, where the loss term for the threshold is the binary entropy. Finally, the loss function of the model quality is the mean squared error (MSE). The total loss is the sum of the losses of the geometric constraint and quality assessment as follows:


(20) \begin{equation*} Loss\left( pred, real\right)={L}_{\mathrm{geometric}}+{L}_{\mathrm{predlDDT}} \end{equation*}

where $pred$ and $real$ are the predicted and real value. Regarding $L$, it corresponds to the respective loss function. In addition, to preserve the model during training, only 4% of the dataset structure is used for validation. For optimization, we utilized the AdamW [45] optimizer with a learning rate of 0.001, which decays at a rate of 1%. The top five models were trained with a batch size of one protein model for 100 epochs, which took ~120 h on a single A100 GPU. For relevant information on neural networks, see Supplementary Table S3.

## RESULTS

We use the constructed structure dataset to train the graph coupled network, which is used to test the non-redundant CASP proteins. Moreover, we participated in the blind test of CAMEO and analyzed the quality assessment data. During the test, the global quality assessment (Global QA) and the accuracy of the local structure quality (Local QA) were used. Local QA describes the quality of each residue, where lDDT is used to evaluate the residue quality. Global QA describes the overall quality of the protein model structure by calculating the mean value of the Local QA. Pearson, Kendall [46], AUC [47], Mean absolute error (MAE), MSE and Top1loss [48] are commonly used evaluation metrics for Global QA. Similarly, Pearson, Spearman [49], Kendall, AUC, MAE and MSE are used as evaluation metrics for Local QA. Pearson estimated the correlation between the predicted and real quality of local residues or overall structure. Greater values indicate a stronger correlation and improved performance of the method. The error between the predicted quality and the real quality was measured using MAE and MSE, with the magnitude of the value indicating the gap from the real quality. A smaller value indicates better performance in predicting the quality. These metrics help assess the performance of models in terms of their accuracy and ability to make predictions.

### Test set construction

The performance of GraphCPLMQA is thoroughly tested on the model quality assessment datasets of CASP13, CASP14 and CASP15. GraphCPLMQA also participated in the continuous blind test of quality evaluation in CAMEO. All the test sets are constructed from the data provided by CASP and CAMEO official websites. The CASP13 and CASP14 test sets (CASP monomer test sets) were constructed by collecting models that were evaluated by all comparison methods and had a sequence similarity of <35% to the proteins in the training set. The CASP13 dataset collects 9390 structural models of 70 individual targets, and the CASP14 dataset collects 9645 models of 69 individual targets. The CASP15 test set (CASP multimer test set) was constructed by collecting structural models whose experimental structures had been released and length did not exceed 3000 residues and collected 9108 models of 34 multimer targets. Our training data (19 November 2021) was constructed before the CASP15, and the impact on the data is completely isolated in time. The CAMEO blind test set consists of 6 months CAMEO data (20 May 2022 to 13 August 2022), which contains 1590 models of 189 proteins. Details of data can be found in Supplementary Tables S4–S6.

### Results on the recent CASP15 multimer test set

**Figure 2:**
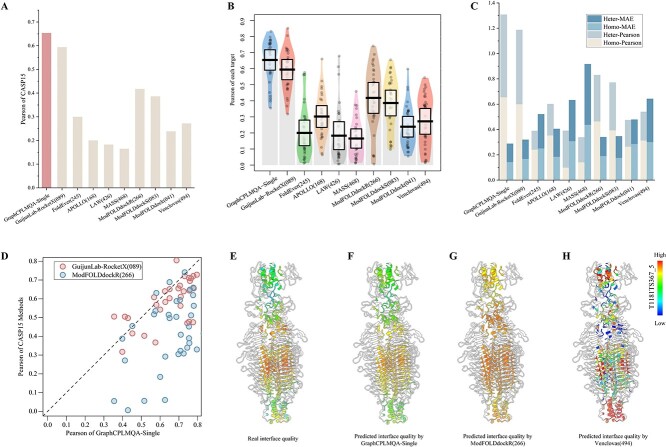
Test results for interface residues in the CASP multimer test set (CASP15). (**A**) The histograms reflect the results of GraphCPLMQA-Single versus other methods of CASP15 on Pearson and MAE. (**B**) The pirate graph shows the Pearson correlation of different methods in predicting the quality of the multimer interface and the quality of the real multimer interface, where the horizontal line is the mean line. (**C**) The histogram depicts the performance analysis of different methods on CASP15 homo-oligomers and hetero-oligomers. (**D**) The scatterplot shows GraphCPLMQA-Single compared with the top method GuijunLab-RocketX and the second method ModFOLDdockR in recent CASP15 interface local quality evaluation. (**E**)–(**H**) For model T1181_TS367_5, different methods predict the quality distribution at the multimer interface.

With the precision breakthrough of Alphafold2 in monomers, research into multimers has become a top priority. Similarly, assessing the quality of multimer interfaces is a future frontier and presents a challenge. The lack of effective MSA information greatly increases the difficulty of predicting and evaluating multimers. However, GraphCPLMQA-Single employs a single-sequence embedding to assess interface quality, as shown in Figure 2 and Supplementary Table S7.

GraphCPLMQA-Single is compared with nine methods in CASP15 for predicting multimer interfaces. On Pearson metrics, GraphCPLMQA-Single has improved by 23.6% compared with ModFOLDdockR [50, 51] (266), and its interface quality prediction ranks second in CASP15 (Supplementary Figure S2A). ModFOLDdockR is a variant optimized for ranking based on ModFOLDdock, which is a multi-model QA server that brings together a series of single-model, clustering and deep learning methods to form a method consensus. On the MAE metric, we observed that our method outperforms GuijunLab-RocketX (089), which is considered one of the top-performing methods for CASP15 multimer evaluation, in terms of predicting interface local quality (Figure 2A). In other metrics, our method also achieves the highest performance with Spearman (0.617), Kendall (0.45), AUC (0.844), MSE (0.035), MAE (0.144), compared with other methods (Table 1). For each target, our method predicts results with higher stability and accuracy than other methods (Supplementary Figure S2B).

**Table 1:** Comparison of GraphCPLMQA-Single with other methods on models of CASP15

Methods	Local interface QA				
	Pearson	Spearman	AUC	MSE	MAE
GraphCPLMA-Single	0.654	0.617	0.844	0.035	0.144
GuijunLab-RocketX	0.594	0.552	0.809	0.048	0.16
FoldEver	0.3	0.289	0.662	0.059	0.2
APOLLO	0.2	0.219	0.544	0.092	0.259
LAW	0.183	0.179	0.433	0.129	0.315
MASS	0.165	0.178	0.443	0.273	0.456
ModFOLDdockR	0.418	0.414	0.737	0.05	0.171
ModFOLDdockS	0.386	0.379	0.724	0.05	0.173
ModFOLDdock	0.239	0.242	0.608	0.097	0.242
Venclovas	0.272	0.286	0.647	0.158	0.32

*Note:* The data come from the CASP15 official website.

We compare with ModFOLDdockR (266) on each target. The results show that prediction accuracy of GraphCPLMQA-Single outperforms ModFOLDdockR (Figure 2D). Evaluation of the model interface for T1181, the predicted quality of GraphCPLMQA-Single is closer to the real quality, where the quality corresponds to the change of color (low: blue, high: red). Furthermore, we analyzed the performance of the evaluation method on different types of multimers (homo-oligomers and hetero-oligomers) as shown in Figure 2C and Supplementary Table S7. Interestingly, the performance of our method on different types of multimers is basically consistent. GraphCPLMQA-Single remains at the highest accuracy for evaluating interfaces in both homo-oligomers and hetero-oligomers.

The above results show that the performance of GraphCPLMQA-Single surpasses other CASP15 methods. Although GraphCPLMQA-Single is trained on monomer data, it performs well on multimer interface evaluation. GraphCPLMQA-Single shows potential for extension to evaluate multimer interfaces, which may be attributed to the following reasons. First, the network has learned the evaluation mode of the local structural quality on proteins; second, the network takes the input protein structure as a whole, regardless of whether it is a multimer or a monomer; finally, the features of the network can describe structural and sequence information of the multimer. However, our method still has deficiencies in the interface evaluation of the CASP15 multimer test set. It can be seen from Supplementary Figure S2D and E that the accuracy of both ends of the abscissa is relatively low, which is arranged from short to long according to the length of the target, and the accuracy of the middle part is relatively high and stable. This shows that the length of the multimer model will have a certain degree of impact on the evaluation accuracy of GraphCPLMQA-Single.

### Results on the CAMEO blind test

**Figure 3:**
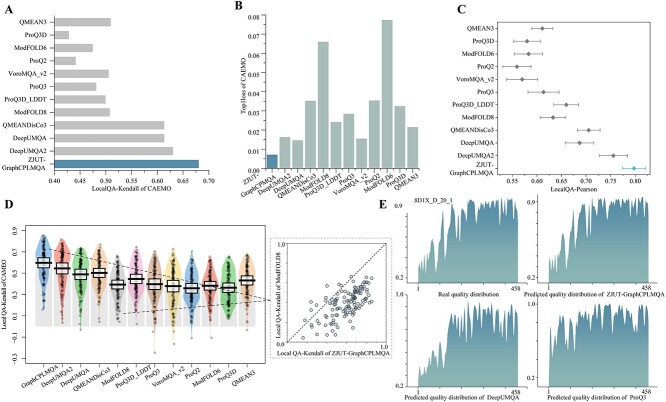
The results of ZJUT-GraphCPLMQA (our server) and other servers on CAMEO blind test (20 May 2022 to 13 August 2022). (**A**, **B**) Histograms depict the results of our method versus other methods on the Kendall and Top1loss metrics. (**C**, **D**) These plots reflect the distribution of results of our method compared with other servers in terms of local indicators of target proteins. Each point in the graph represents the statistical results of all models for a protein target. (**C**) The diamond is the mean and the range of confidence interval is 0.9. (**D**) The black horizontal line is the mean and the range of the standard deviation is 0.3. (**E**) On protein model 8D1X_D_20_1, real quality distribution versus predicted distribution for other servers.

We developed the server ZJUT-GraphCPLMQA (server 46) based on the GraphCPLMQA method to participate in CAMEO-QE. In addition, more than 3134 protein models have been evaluated. In the competition, other participating servers included DeepUMQA2 [22], DeepUMQA [20], QMEANDisCo3 [9], ProQ3D_LDDT [23], VoroMQA_v2,QMEAN3 [52], ProQ2 [53], ModFOLD8 [8], ProQ3D [23], ModFOLD6 [54], VoroMQA_sw5 [18]. We download the test data on the CAMEO official website (from 20 May 2022 to 13 August 2022). Among the 128, 018 residues in the CAMEO blind test, the evaluation accuracies of GraphCPLMQA for local residuals are Pearson (0.891), Kendall (0.680), AUC (0.942) and MAE (0.081), all of which exceed the accuracies of other servers, and MSE (0.015) is inferior to DeepUMQA2 (Table 2). The local Pearson and Kendall distributions of target proteins are shown in Figure 3C and D. In the Global QA, the Pearson and AUC accuracy of GraphCPLMQA are 0.924 and 0.967, higher than QMEANDisCo 3, and the accuracy of MAE (0.077) is second only to DeepUMQA2. An analysis was conducted on the quality predictions of different servers for the protein model 8D1X_D_20_1. The quality distribution predicted by our server and the real quality trend are shown in Figure 3E. Furthermore, in Supplementary Figure S3A–D, the model quality corresponds to the change of color, and it can be clearly seen that the accuracy of our prediction is higher. There are other models of case in Supplementary Figure S4, and blind test results in the Supplementary Figure S5.

**Table 2:** Results of ZJUT-GraphCPLMQA (server 46) on CAMEO blind test set (from 20 May 2022 to 13 August 2022)

Methods	Local QA					Global QA				
	Person	Kendall	AUC	MSE	MAE	Person	Kendall	AUC	MAE	Top1loss
ZJUT-GraphCPLMA	0.891	0.680	0.942	0.015	0.081	0.924	0.741	0.967	0.077	0.007
DeepUMQA2	0.878	0.630	0.941	0.013	0.083	0.908	0.687	0.966	0.066	0.016
DeepUMQA	0.835	0.613	0.923	0.017	0.099	0.874	0.684	0.956	0.074	0.015
QMEANDisCo3	0.829	0.613	0.925	/	/	0.885	0.664	0.962	0.354	0.035
ModFOLD8	0.771	0.507	0.894	0.026	0.127	0.824	0.557	0.920	0.094	0.066
ProQ3D_LDDT	0.769	0.499	0.887	0.038	0.151	0.824	0.530	0.904	0.137	0.024
ProQ3	0.749	0.481	0.881	0.039	0.156	0.825	0.559	0.914	0.129	0.028
VoroMQA_v2	0.733	0.505	0.880	0.051	0.176	0.824	0.584	0.926	0.166	0.015
ProQ2	0.717	0.441	0.859	0.050	0.176	0.812	0.520	0.900	0.159	0.035
ModFOLD6	0.716	0.474	0.877	0.052	0.182	0.811	0.536	0.906	0.160	0.077
ProQ3D	0.708	0.428	0.853	0.037	0.151	0.811	0.509	0.892	0.112	0.032
QMEAN3	0.705	0.509	0.886	0.165	0.333	0.807	0.611	0.921	0.357	0.021
VoroMQ_sw5	0.596	0.395	0.828	0.051	0.188	0.766	0.515	0.878	0.160	0.023

*Note:* ZJUT-GraphCPLMQA is developed based on the GraphCPLMQA.

### Results on the CASP monomer test set

**Figure 4:**
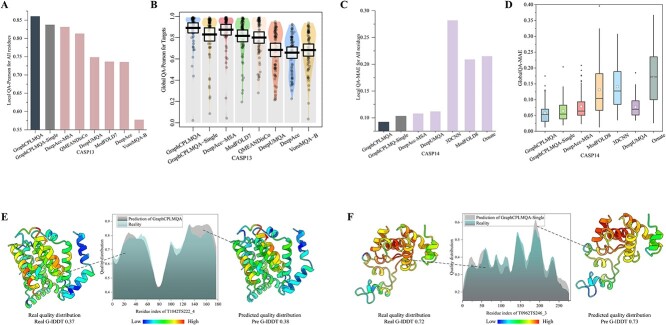
Performance comparison between GraphCPLMQA and other methods on the CASP monomer test set. (**A**) For the all residues of CASP13 monomer test set, GraphCPLMQA and GraphCPLMQA-Single were compared with other methods based on the Pearson correlation between the predicted and real quality of residues. (**B**) The pirate graph reflects the comparison results of the global indicator Pearson on CASP13 where the horizontal bar is the mean line. (**C**) For the all residues of CASP14 monomer test set, GraphCPLMQA and GraphCPLMQA-Single were compared with other methods based on the MAE between the predicted and real quality of residues. (**D**) In the boxplot, the horizontal line is the median, and the box is the mean. (**E**, **F**) The predictions are compared with the true quality results.

For 9390 protein models of CASP13 and 9645 protein models of CASP14, GraphCPLMQA and GraphCPLMQA-Single compare performance with state-of-the-art methods on the CASP monomer dataset (Figure 4, Supplementary Figure S6). In the monomer test set, GraphCPLMQA achieves the highest accuracy on both Global QA and Local QA metrics, surpassing other comparable methods. Among the compared methods, QMEANDisCo [9] and DeepAccNet-MSA were one of the best-performing model quality assessment methods in CASP13 and CASP14, respectively. GraphCPLMQA is analyzed using Pearson and MAE with QMEANDisCo and DeepAccNet-MSA for residues of all models. In terms of global quality, GraphCPLMQA had a Pearson of 0.927, representing a 3% improvement over DeepAccNet-MSA (Supplementary Tables S8 and S9). We analyzed the quality distribution of the T1042 and T0962 models, and the results predicted by GraphCPLMQA are basically the same as the real quality, where the range of color is the reference real quality distribution (Figure 4E and F). There are other models of case in Supplementary Figure S7. GraphCPLMQA-Single uses a single-sequence embedding for comparison with methods that use single-sequence information. Similarly, GraphCPLMQA-Single outperforms other methods using single-sequence information on all metrics, such as DeepUMQA, QMEANDisCo, ModFOLD8, DeepAccNet, etc. GraphCPLMQA-Single shows similar performance to DeepAccNet-MSA using MSA. In addition, the scatterplots of GraphCPLMQA and GraphCPLMQA-Single compared with other methods in Supplementary Figures S8–S15. The above results show that embeddings from language models and graph-coupled networks improve the accuracy of model quality assessment. The impact of different parts on the accuracy of the method can be seen in the ablation studies.

### Ablation studies

**Figure 5:**
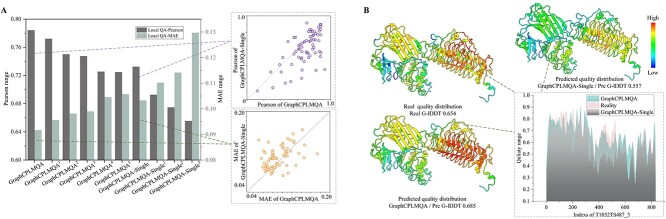
The impact of various components on the performance of GraphCPLMQA in CASP monomer test set. (**A**) Variation of network architecture and features are on the overall performance of our method. (**B**) Prediction results of GraphCPLMQA and GraphCPLMQA-Single on T1052 monomer model. The real quality distribution range is as standard.

The impact of the features and network architecture for GraphCPLMQA and GraphCPLMQA-Single on the non-redundant CASP monomer test datasets were analyzed (Figure 5, Supplementary Figure S16, Tables S10–S13). At the sequence feature level, we compare the performance of GraphCPLMQA using MSA embedding with GraphCPLMQA-single using single sequence embedding. In terms of various evaluation metrics, the performance of GraphCPLMQA with MSA embedding was superior to that of the counterpart without MSA (Figure 5A). This suggests that the MSA contains richer structural information compared with single sequence. The embeddings derived from MSA provide better guidance for evaluating model quality. Furthermore, we analyze in detail the impact of components on method performance below.

To investigate the impact of the components, we modified the full version of GraphCPLMQA by removing some features and changing the network architecture. Different network models were retrained to test the results and analyze the effect of these modifications. First, GraphCPLMQA^1^ was created by replacing the transformer strategy inverted bottleneck of GraphCPLMQA with residual block in the decoding module, and GELU [42] was replaced with ReLU [55] to create GraphCPLMQA^1^. Regarding local metrics, there was a varying degree of decline in the prediction accuracy, while there was no significant change observed in global metrics. This indicated that the transformer strategy could further capture the local structural information. Second, the structural embeddings were removed from the language model in GraphCPLMQA^1^, leading to a decline in performance for GraphCPLMQA^2^. The high-dimensional structure features may imply some properties of protein structure that contribute to better learning of the network. Then, we changed the output mode of the encoding module (GraphCPLMQA^3^) and the connection architecture between modules (GraphCPLMQA^4^) on the GraphCPLMQA^2^ model. These operations may relatively weaken the sequence–structure relationship in the encoding module so that the encoding result has an impact on the decoding structure–quality relationship. Finally, based on GraphCPLMQA^2^ model, the triangle position and residual-level contact order features were removed (GraphCPLMQA^5^). The reduction in accuracy on the local metrics implies that these characteristics can supplement the portrayal of the local structure.

On the GraphCPLMQA-Single model, we analyze the effect of different single sequence language models on the performance of the method. Specifically, the network models GraphCPLMQA-Single^1^ and GraphCPLMQA-Single^2^ were retrained with the high-dimensional sequence embedding of the ESM-1b [56] and ESM-1v [57] language models, respectively. The use of these embeddings resulted in a noteworthy decrease in performance. Furthermore, the input pattern of sequence embedding was explored using the GraphCPLMQA-Single^3^ network model. GraphCPLMQA-Single^3^ used the sequence embeddings of all layers of the ESM-1v language model by taking the mean. Although GraphCPLMQA-Single^3^ is based on GraphCPLMQA-Single^2^ using ESM-1v embedding, the results still show that this approach introduces significant noise that may affect the accuracy of the predictions. For GraphCPLMA-Single^4^, we removed the loss of geometric constraints and retrained the network model. The performance of the method decreased on all metrics, indicating that geometric constraints may potentially guide local accuracy evaluation.

### Compared with AlphaFold2

For the results of AlphaFold2 prediction in CASP14, we used the official website code of AlphaFold2 (https://github.com/deepmind/alphafold) to predict 69 sequences of CASP14. AlphaFold2 produced five output models for each sequence, resulting in a total of 345 models with pLDDT. GraphCPLMQA and GraphCPLMQA-Single assessed each model quality of AlphaFold2, respectively. The quality of GraphCPLMQA assessment exceeds the self-assessment of AlphaFold2 on MAE (Supplementary Figure S17). Out of the 345 AlphaFold2 models, 253 evaluated results exceeded AlphaFold2 pLDDT. On the 207 AlphaFold2 structures without template information, GraphCPLMQA had 150 better evaluated results. GraphCPLMQA-Single performed slightly better than AlphaFold2 pLDDT on all structures, including those without a template. In addition, the ability to select AlphaFold2 models was also evaluated (Supplementary Figure S17B). Specifically, for the AlphaFold2 dataset, the best structure was selected from the five predicted models of AlphaFold2 and compared with the best predicted model (rank_0) of AlphaFold2. On the test set, we had 26 structures in lDDT better than the best structure of AlphaFold2. Although the gap with AlphaFold2’s selection model is small, this shows that GraphCPLMQA has reached the accuracy of AlphaFold2’s selection in model quality assessment, and AlphaFold2 can only evaluate and select the model it predicts.

**Figure 6:**
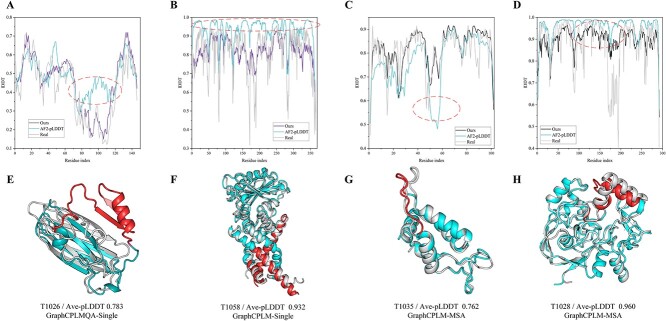
Results of our evaluation of AlphaFold2 structures compared with the AlphaFold2 pLDDT self-assessment on AlphaFold2 dataset. (**A**–**D**) Line graphs correspond to different AlphaFold2 models, and the graphs contain the results of our evaluation, the pLDDT of AlphaFold2 and the real lDDT. (**E**–**H**) Gray represents the native structure, sky blue is the structure of AlphaFold2 and red represents misfolding.

Furthermore, we analyzed the evaluation of GraphCPLMQA on AlphaFold2 medium and high precision models versus its self-evaluation. Figure 6A–D corresponds to Figure 6E–H where gray represents the real structure, sky blue is the structure of AlphaFold2 and red represents the region with relatively large folding error. On the AlphaFold2 structure with medium quality (Figure 6A, C), we could basically predict the distribution of local quality and it was very close to the real distribution. The predicted local AlphaFold2 structure is not consistent with the native structure. In the case of AlphaFold2 pLDDT, it is possible that AlphaFold2 is not precise in local quality assessment, or even results in an opposite assessment, as indicated by the red area. This mets that the accuracy of AlphaFold2 local structure prediction is closely related to the evaluation of local structure. To some extent, the pLDDT of AlphaFold2 may not reflect the quality of the local structure. For the high-quality AlphaFold2 structure (Figure 6B, D), our evaluation results were more consistent with the distribution of the real quality. However, most predicted results of AlphaFold2 were higher than the real quality. The results show that our method helps to complement the deficiencies that exist in the pLDDT of AlphaFold2. In future studies, GraphCPLMQA may also provide a valuable reference for predicting models in AlphaFoldDB that do not have native structures.

## CONCLUSION

In this study, we propose GraphCPLMQA, a novel approach for evaluating model quality that combines graph coupled networks and embeddings from protein language models. GraphCPLMQA utilizes sequence and structure embeddings, as well as additional model features, to establish the relationship among sequence, structure and quality. By predicting protein model quality scores, GraphCPLMQA outperforms other state-of-the-art assessment methods in terms of accuracy on the CASP15, CASP13, CASP14 and CAMEO test sets. GraphCPLMQA also achieves excellent results in the continuous evaluation of CAMEO-QE.

**Key Points**

- In this study, we propose GraphCPLMQA, a novel approach for evaluating residue-level (local) model quality that combines graph coupled networks and embeddings from protein language models.
- We design a graph-coupled network based on an encoder-decoder module to establish a potential mapping relationship between sequence, structure and quality, which takes full advantage of the high-dimensional embedding of protein language models.
- To describe the protein structure and its complexity, we designed the triangle location feature and residue-level contact order.
- Compared with CASP15 local/per-residue interface evaluation methods, GraphCPLMQA using single-sequence embeddings achieved the best performance among 9108 models in the local residue interface test set of CASP15 multimers. In CAMEO blind test (20 May 2022 to 13 August 2022), GraphCPLMQA ranked first compared with other servers (https://www.cameo3d.org/quality-estimation). GraphCPLMQA also outperforms state-of-the-art methods on 19 035 models in CASP13 and CASP14 monomer test set.

## Supplementary Material

Supplementary_bbad420

All_Data_bbad420

## Data Availability

The authors declare that the data supporting the findings and conclusions of this study are available within the paper and its Supplementary Information file. Other data are available from the corresponding author upon reasonable requests. The GraphCPLMQA server and source code are available for free at http://zhanglab-bioinf.com/GraphCPLMQA/ and https://github.com/iobio-zjut/GraphCPLMQA, respectively.

## Notes

**Dong Liu** is a PhD candidate in the College of Information Engineering, Zhejiang University of Technology. His research interests include bioinformatics, intelligent information processing, the development of machine learning models and algorithms.

**Biao Zhang** is a lecturer in the College of Information Engineering, Zhejiang University of Technology. His research interests include bioinformatics, intelligent information processing and optimization theory.

**Jun Liu** is a PhD candidate in the College of Information Engineering, Zhejiang University of Technology. His research interests include bioinformatics, intelligent information processing and optimization theory.

**Hui Li** is a researcher of AI in the BioMap. His research interests include the development of machine learning models and algorithms and bioinformatics.

**Le Song** is a Chief Scientist of AI in the BioMap & MBZUAI. His research interests include the development of machine learning models and algorithms and bioinformatics.

**Guijun Zhang** is a professor in the College of Information Engineering, Zhejiang University of Technology. His research interests include bioinformatics, intelligent information processing and optimization theory.
