# Supplementary material for: Assessing protein model quality based on deep graph coupled networks using protein language model
Source: Brief Bioinform. 2023 Nov 28;25(1):bbad420. doi: 10.1093/bib/bbad420 (PMC10685403; doi:10.1093/bib/bbad420)
Supplement: Supplementary_bbad420 [file supplementary_bbad420.zip › Supplementary_bbad420.pdf]

Supplementary Information for

GraphCPLMQA: Assessing protein model quality based on deep graph coupled networks using protein language model

Dong Liu†, Biao Zhang†, Jun Liu, Hui Li*, Le Song* and Gui-Jun Zhang*

This PDF file includes:

- Supplementary figures S1 to S17
- Supplementary tables S1 to S13
- Supplementary text S1 to S4

Supplementary figures

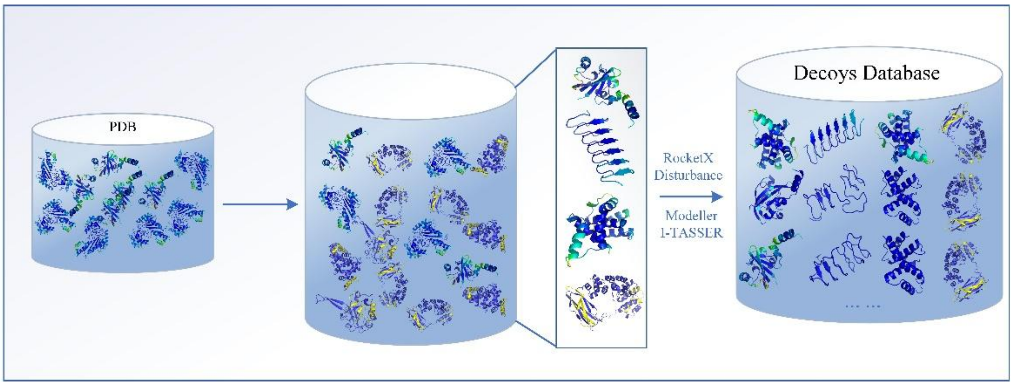

Supplementary Figure S1 Schematic diagram of data set process construction

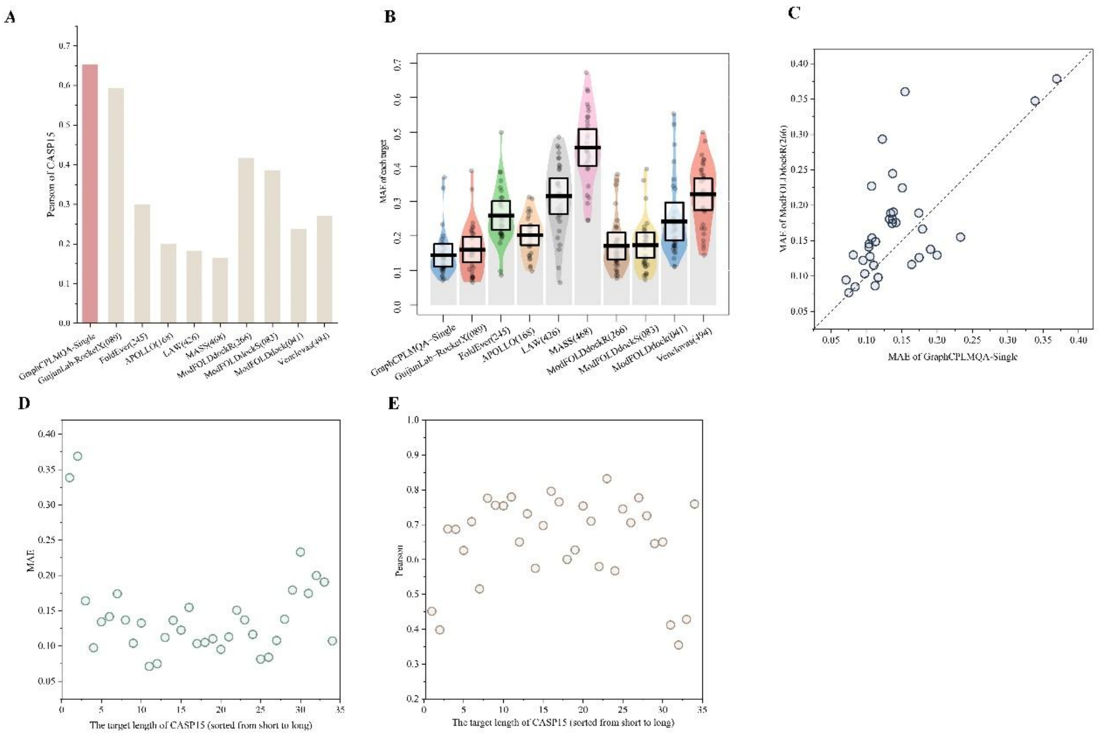

Supplementary Figure S2 Results on the CASP complex test set

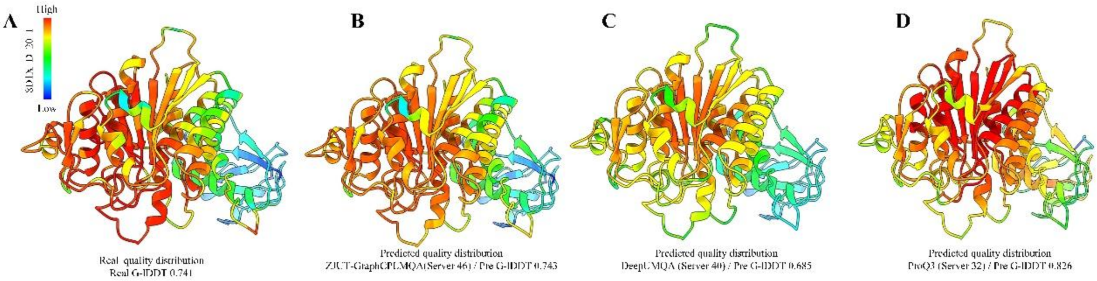

Supplementary Figure S3 Result on CAMEO blind test

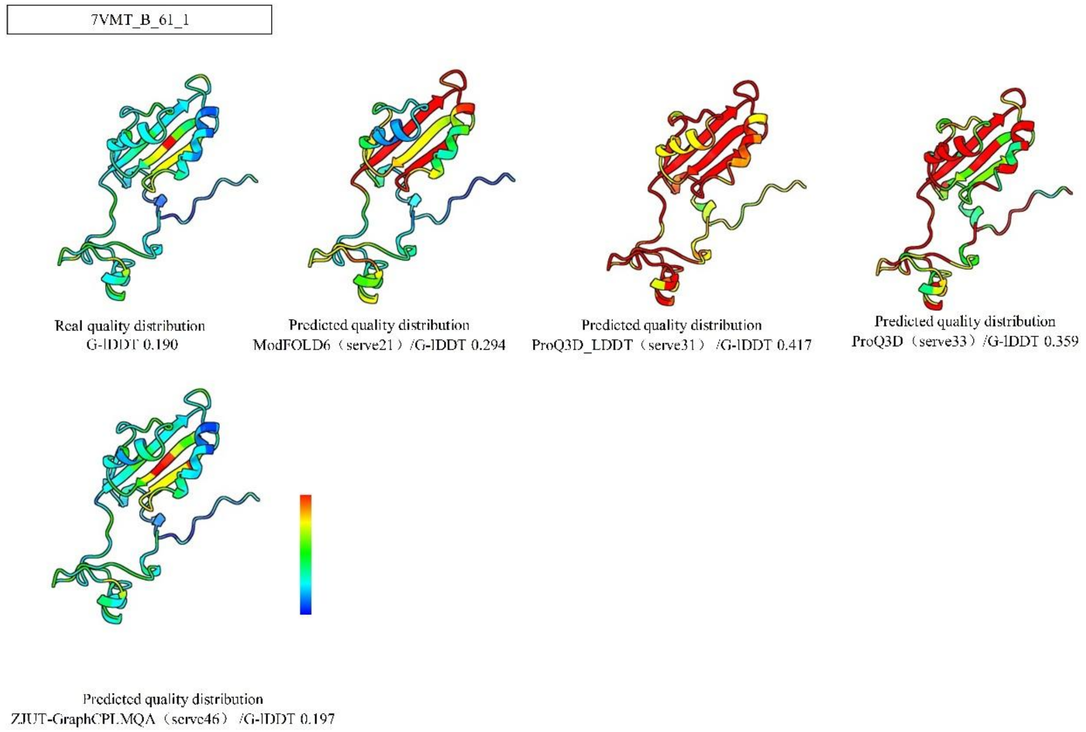

Supplementary Figure S4 The prediction of ZJUT-GraphCPLMQA on the model of CAMEO dataset

6-months [2022-05-13 - 2022-11-05] - "All" dataset

Predictor Name	Predictor Type	Avg. response time (hh:mm:ss)	Structural models			ROC		ROC^{normalized}		PR		PR^{normalized}	
			#Submitted	#Received	%	$AUC_{0,1}$	$AUC^*_{0,0.2}$	$AUC_{0,1}$	$AUC^*_{0,0.2}$	$AUC_{0,1}$	$AUC^*_{0.8,1}$	$AUC_{0,1}$	$AUC^*_{0.8,}$
ZJUT-GraphCPLMQA	server	38:01:35	3262	3136	96.1	0.95	0.85	0.91	0.81	0.92	0.67	0.89	0.65
DeepUMQA2	server	28:20:30	3262	3131	96.0	0.94	0.81	0.90	0.78	0.90	0.65	0.87	0.62
ModFOLD9	server	13:08:40	3262	2819	86.4	0.93	0.78	0.80	0.67	0.89	0.59	0.77	0.51
DeepUMQA	server	03:28:26	3262	2561	78.5	0.93	0.75	0.73	0.59	0.87	0.61	0.68	0.48
Atom_ProteinQA	server	00:02:06	83	83	100.0	0.93	0.71	0.93	0.71	0.87	0.66	0.87	0.66
QMEANDisCo 3	server	00:01:19	3262	3259	99.9	0.91	0.69	0.91	0.69	0.83	0.56	0.83	0.56
ProQ3D_LDDT	server	28:29:46	3262	2845	87.2	0.89	0.66	0.78	0.57	0.82	0.53	0.72	0.46
ProQ3	server	28:32:46	3262	2842	87.1	0.88	0.65	0.77	0.57	0.81	0.50	0.71	0.44
QMEAN 3	server	00:00:24	3262	3259	99.9	0.88	0.64	0.88	0.64	0.79	0.47	0.79	0.47
VoroMQA_v2	server	00:00:10	3262	3262	100.0	0.88	0.63	0.88	0.63	0.79	0.49	0.79	0.49

CAMEO result (6 months from 2022-05-13~2022-11-05)

3-months [2022-08-12 - 2022-11-05] - "All" dataset

Predictor Name	Predictor Type	Avg. response time (hh:mm:ss)	Structural models			ROC		$ROC^{normalized}$		PR		$PR^{normalized}$	
			#Submitted	#Received	%	$AUC_{0,1}$	$AUC^{*}_{0,0.2}$	$AUC_{0,1}$	$AUC^{*}_{0,0.2}$	$AUC_{0,1}$	$AUC^{*}_{0.8,1}$	$AUC_{0,1}$	$AUC^{*}_{0.8,1}$
ZJUT-GraphCPLMQA	server	42:58:28	1745	1691	96.9	0.95	0.86	0.93	0.84	0.93	0.69	0.90	0.67
DeepUMQA2	server	34:17:15	1745	1743	99.9	0.95	0.83	0.95	0.83	0.91	0.66	0.91	0.66
ModFOLD9	server	15:38:08	1745	1402	80.3	0.93	0.79	0.75	0.64	0.89	0.59	0.71	0.47
DeepUMQA	server	06:18:43	1745	1404	80.5	0.93	0.77	0.75	0.62	0.87	0.60	0.70	0.48
ProQ3D_LDDT	server	30:16:01	1745	1460	83.7	0.91	0.69	0.76	0.58	0.84	0.56	0.70	0.47
QMEANDisCo 3	server	00:01:24	1745	1745	100.0	0.91	0.68	0.91	0.68	0.82	0.54	0.82	0.54
ProQ3	server	30:18:35	1745	1458	83.6	0.90	0.68	0.75	0.57	0.83	0.52	0.69	0.44
QMEAN 3	server	00:00:27	1745	1745	100.0	0.89	0.67	0.89	0.67	0.80	0.48	0.80	0.48
VoroMQA_v2	server	00:00:12	1745	1745	100.0	0.90	0.66	0.90	0.66	0.81	0.50	0.81	0.50
ProQ2	server	00:11:29	1745	1744	99.9	0.87	0.62	0.87	0.62	0.77	0.44	0.77	0.44

CAMEO result (3 months from 2022-08-12~2022-11-05)

1-month [2022-10-14 - 2022-11-05] - "All" dataset

Predictor Name	Predictor Type	Avg. response time (hh:mm:ss)	Structural models			ROC		$ROC^{normalized}$		PR		$PR^{normalized}$	
			#Submitted	#Received	%	$AUC_{0,1}$	$AUC^{*}_{0,0.2}$	$AUC_{0,1}$	$AUC^{*}_{0,0.2}$	$AUC_{0,1}$	$AUC^{*}_{0.8,1}$	$AUC_{0,1}$	$AUC^{*}_{0.8,1}$
ZJUT-GraphCPLMQA	server	45:08:26	536	533	99.4	0.94	0.83	0.94	0.83	0.90	0.63	0.90	0.62
DeepUMQA2	server	37:33:52	536	536	100.0	0.94	0.78	0.94	0.78	0.87	0.61	0.87	0.61
DeepUMQA	server	20:05:24	536	428	79.9	0.91	0.70	0.72	0.56	0.81	0.50	0.65	0.40
ModFOLD9	server	10:25:21	536	306	57.1	0.90	0.69	0.52	0.39	0.79	0.43	0.45	0.25
QMEANDisCo 3	server	00:01:24	536	536	100.0	0.91	0.66	0.91	0.66	0.80	0.53	0.80	0.53
QMEAN 3	server	00:00:25	536	536	100.0	0.89	0.65	0.89	0.65	0.78	0.46	0.78	0.46
ProQ3D_LDDT	server	33:22:40	536	421	78.5	0.89	0.62	0.70	0.49	0.80	0.53	0.63	0.41
ProQ3	server	33:26:37	536	422	78.7	0.87	0.62	0.69	0.49	0.79	0.48	0.62	0.38
VoroMQA_v2	server	00:00:08	536	536	100.0	0.88	0.60	0.88	0.60	0.76	0.45	0.76	0.45
ProQ2	server	00:21:16	536	536	100.0	0.86	0.59	0.86	0.59	0.74	0.41	0.74	0.41

CAMEO result (1 month from 2022-10-14~2022-11-05)

1-week [2022-12-03] - "All" dataset

Predictor Name	Predictor Type	Avg. response time (hh:mm:ss)	Structural models			ROC		$ROC^{normalized}$		PR		$PR^{normalized}$	
			#Submitted	#Received	%	$AUC_{0,1}$	$AUC^{*}_{0,0.2}$	$AUC_{0,1}$	$AUC^{*}_{0,0.2}$	$AUC_{0,1}$	$AUC^{*}_{0.8,1}$	$AUC_{0,1}$	$AUC^{*}_{0.8,1}$
ZJUT-GraphCPLMQA	server	42:12:43	141	140	99.3	0.90	0.73	0.89	0.73	0.84	0.45	0.84	0.45
ModFOLD9	server	08:01:05	141	115	81.6	0.88	0.68	0.71	0.56	0.81	0.42	0.66	0.35
DeepUMQA2	server	40:39:15	141	141	100.0	0.86	0.64	0.86	0.64	0.77	0.38	0.77	0.38
DeepUMQA	server	00:27:15	141	50	35.5	0.87	0.64	0.31	0.23	0.84	0.55	0.30	0.19
QMEANDisCo 3	server	00:02:21	141	141	100.0	0.86	0.59	0.86	0.59	0.73	0.42	0.73	0.42
QMEAN 3	server	00:00:29	141	141	100.0	0.85	0.57	0.85	0.57	0.71	0.41	0.71	0.41
VoroMQA_v2	server	00:00:09	141	141	100.0	0.85	0.55	0.85	0.55	0.71	0.40	0.71	0.40
ProQ3D_LDDT	server	31:51:17	141	130	92.2	0.85	0.54	0.78	0.50	0.70	0.44	0.64	0.40
ProQ3	server	31:54:48	141	130	92.2	0.85	0.53	0.78	0.49	0.71	0.43	0.66	0.39
ProQ3D	server	31:58:58	141	130	92.2	0.81	0.46	0.75	0.42	0.63	0.38	0.58	0.35
ModFOLD6	server	41:00:47	141	136	96.5	0.79	0.44	0.77	0.43	0.63	0.35	0.61	0.34
ModFOLD8	server	03:14:03	141	128	90.8	0.80	0.44	0.73	0.40	0.63	0.36	0.57	0.33

CAMEO result (1 week from 2022-11-26~2022-12-03)

Supplementary Figure S5

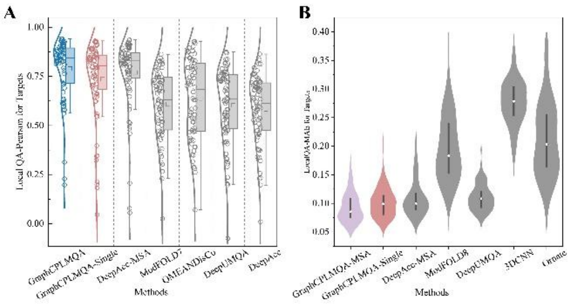

Supplementary Figure S6 GraphCPLMQA compared to other methods on CASP monomer test set

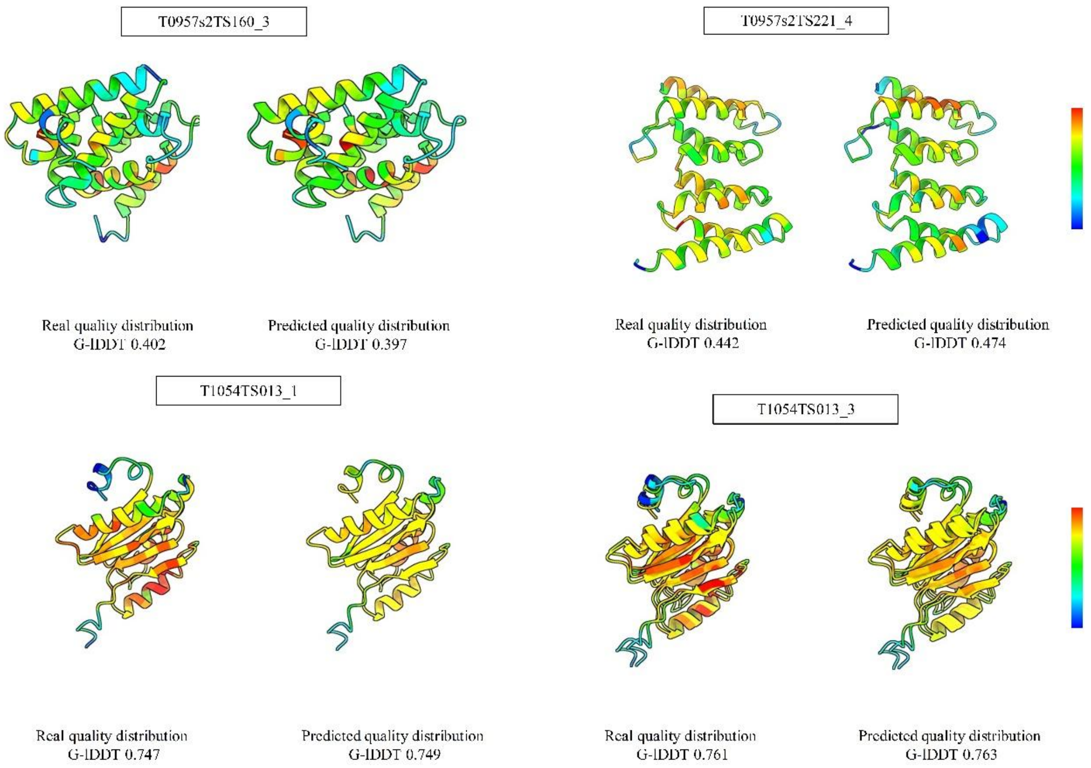

Supplementary Figure S7 The prediction of GraphCPLMQA on the model of CASP dataset

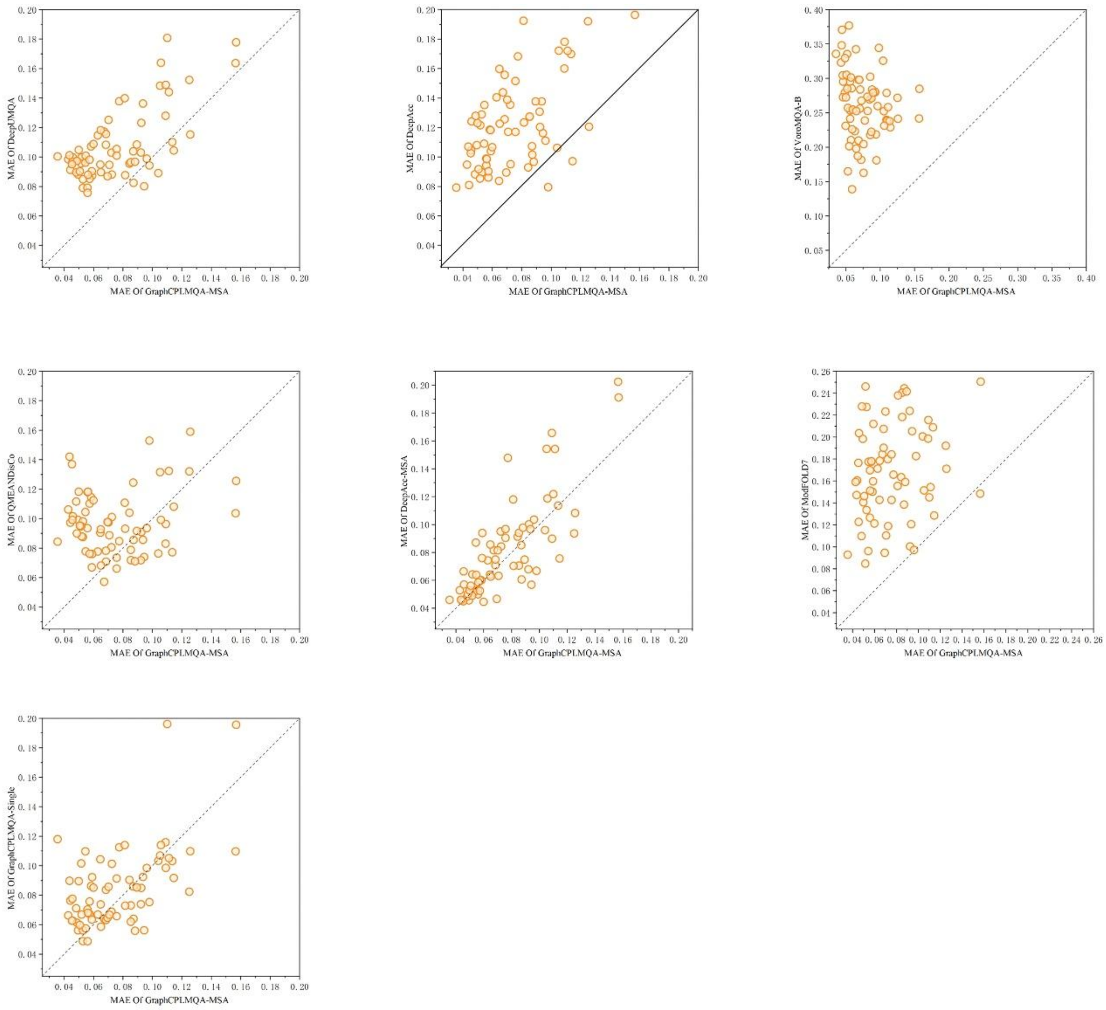

Supplementary Figure S8 MAE of GraphCPLMQA (GraphCPLMQA-MSA) compared to other methods on CASP13

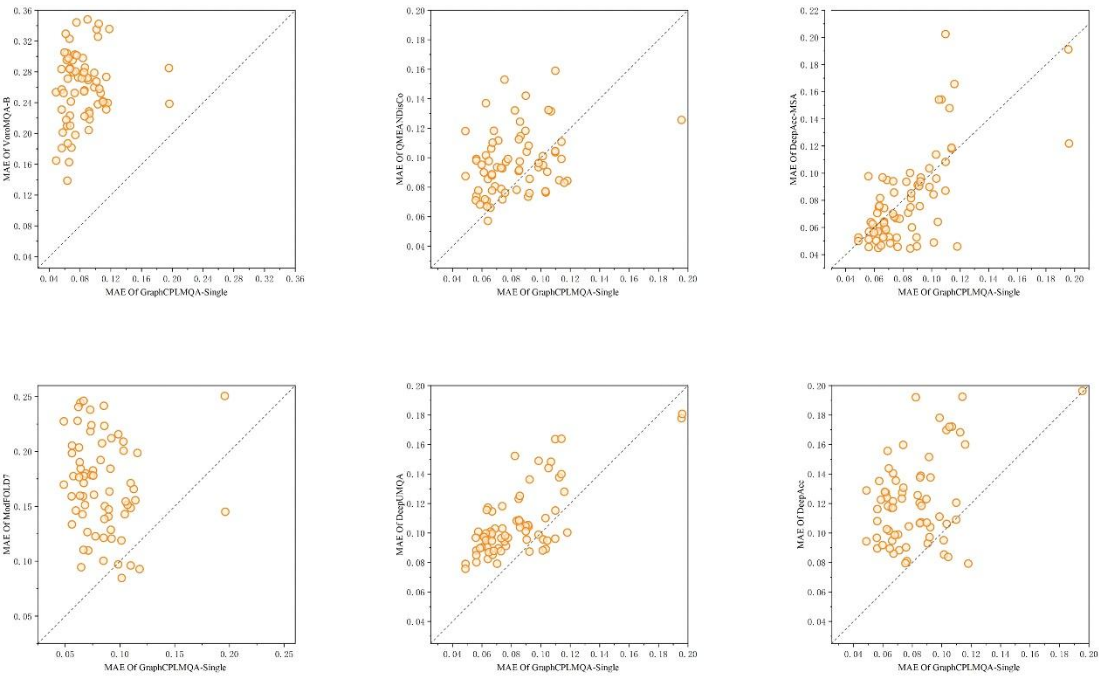

Supplementary Figure S9 MAE of GraphCPLMQA-Single compared to other methods on CASP13.

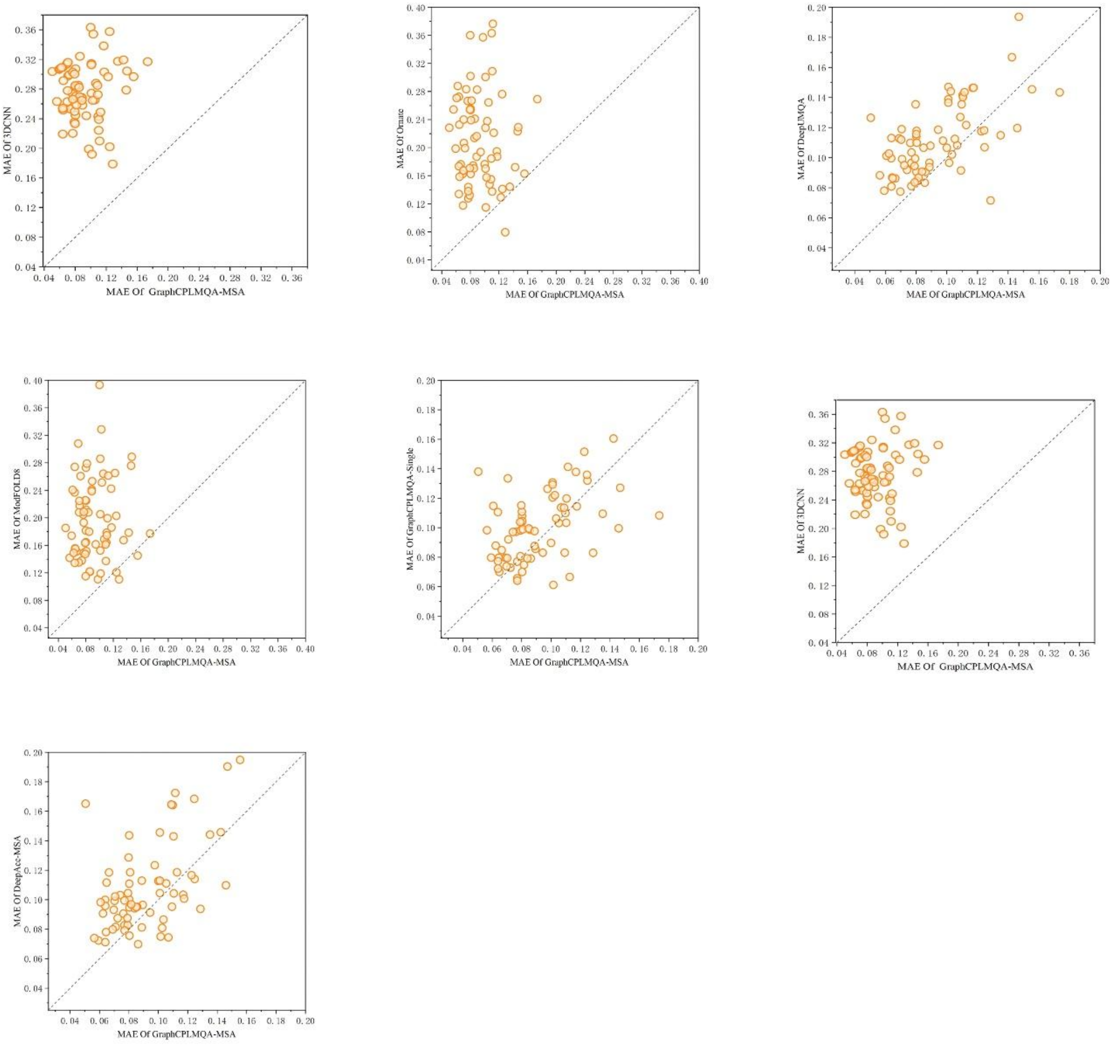

Supplementary Figure S10 MAE of GraphCPLMQA (GraphCPLMQA-MSA) compared to other methods on CASP14

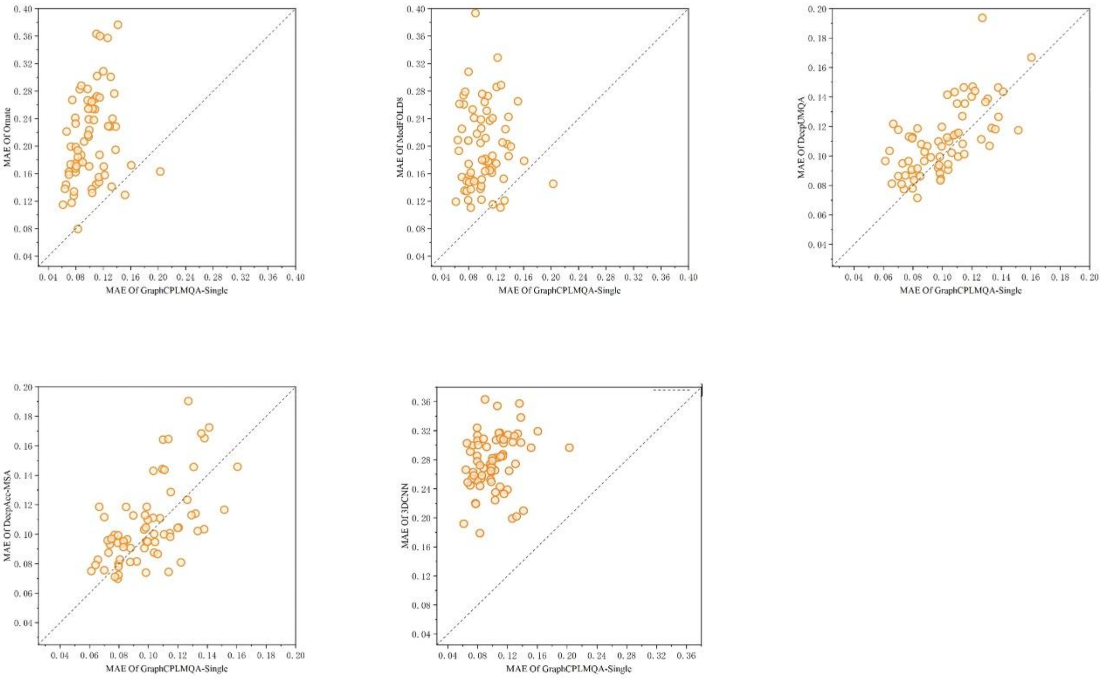

Supplementary Figure S11 MAE of GraphCPLMQA-Single compared to other methods on CASP14

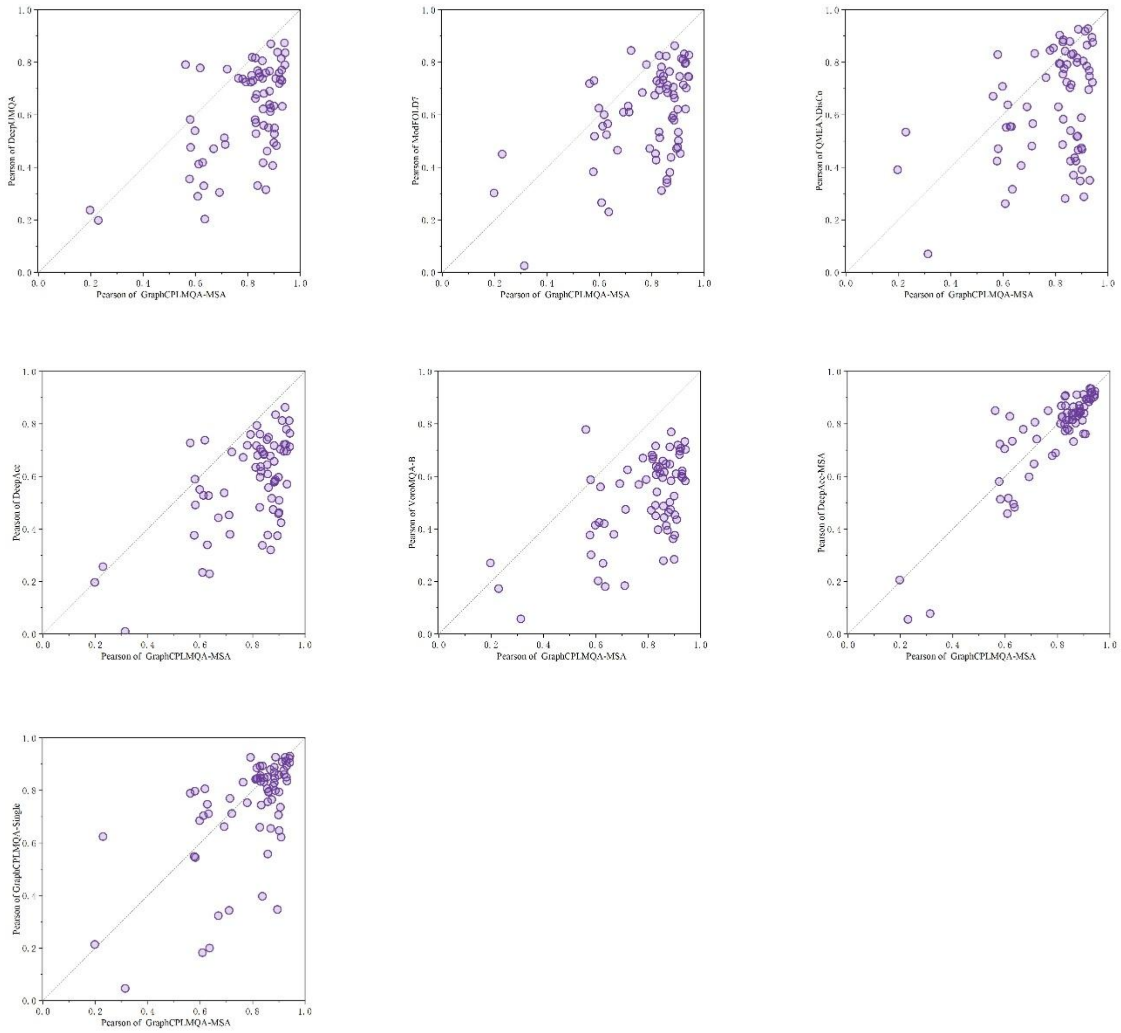

Supplementary Figure S12 Pearson of GraphCPLMQA (GraphCPLMQA-MSA) compared to other methods on CASP13

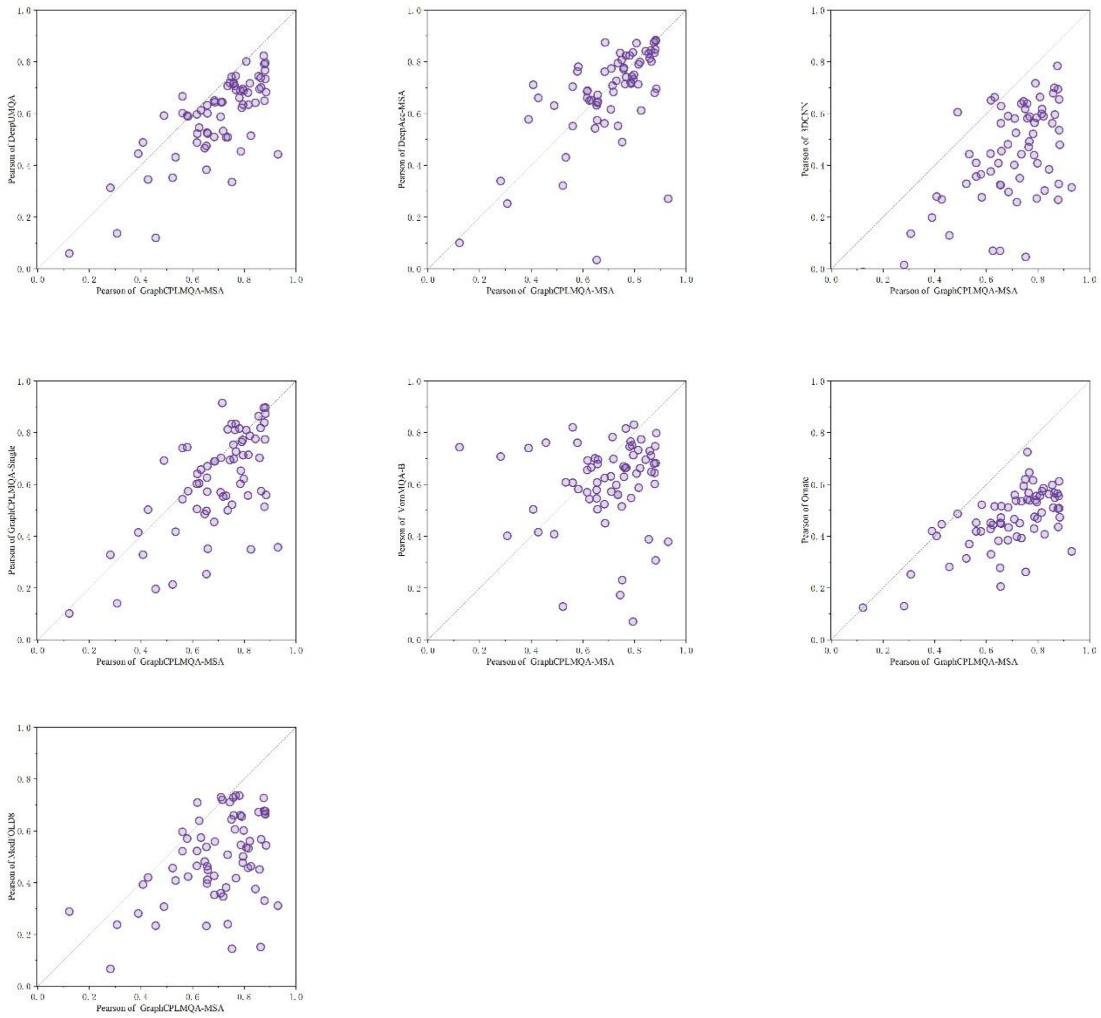

Supplementary Figure S13 Pearson of GraphCPLMQA (GraphCPLMQA-MSA) compared to other methods on CASP14

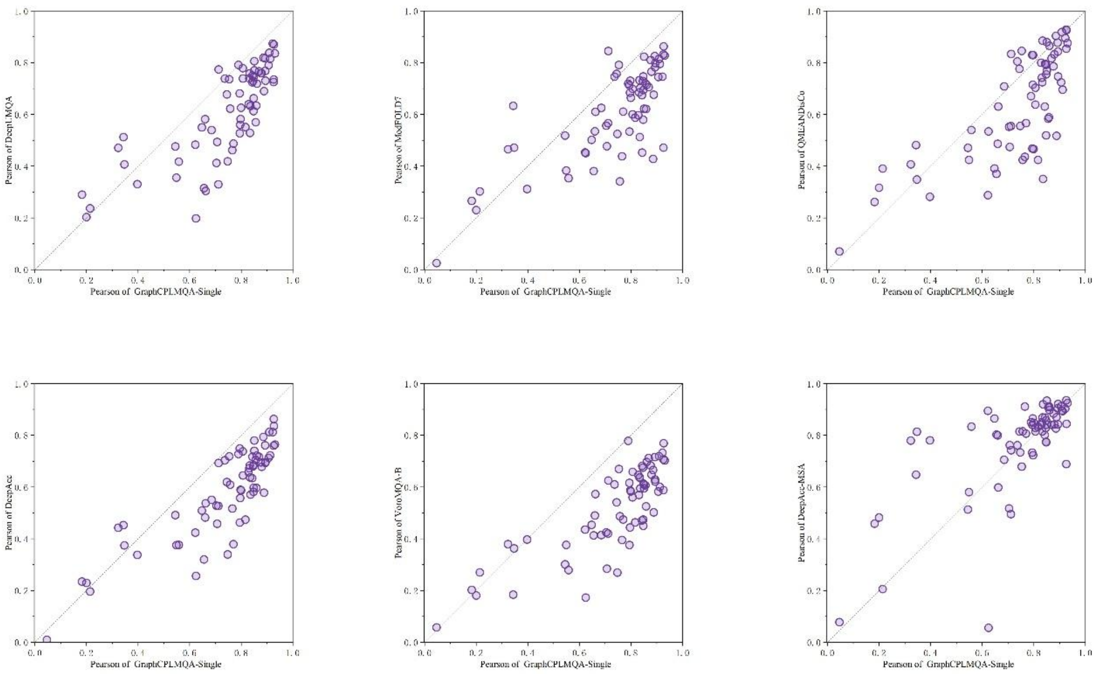

Supplementary Figure S14 Pearson of GraphCPLMQA-Single compared to other methods on CASP13

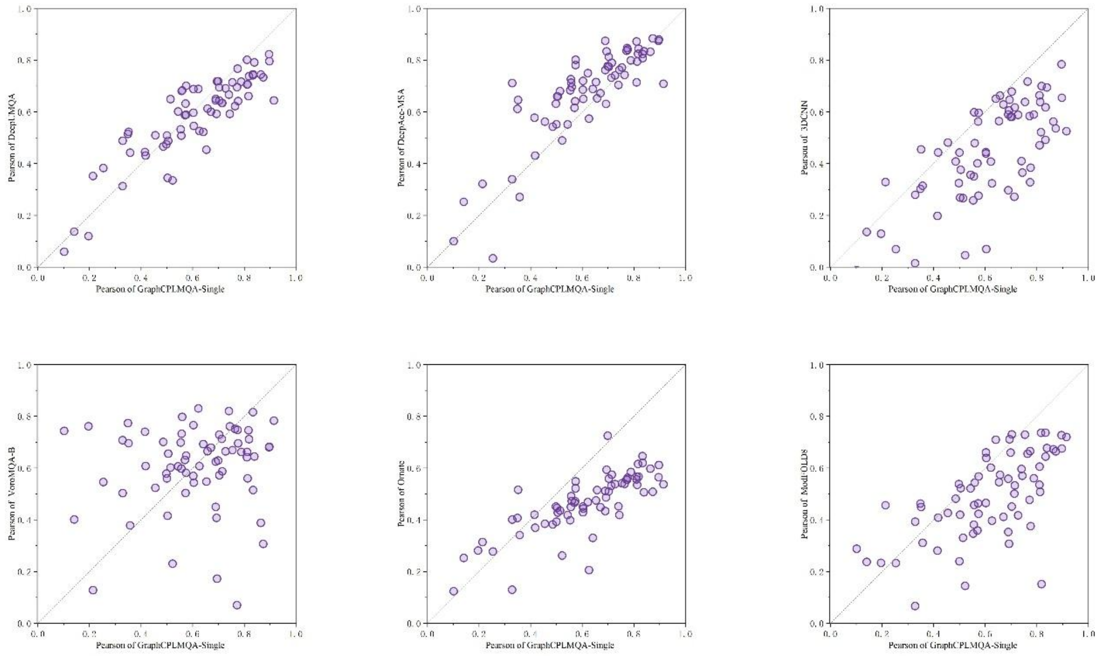

Supplementary Figure S15 Pearson of GraphCPLMQA-Single compared to other methods on CASP14

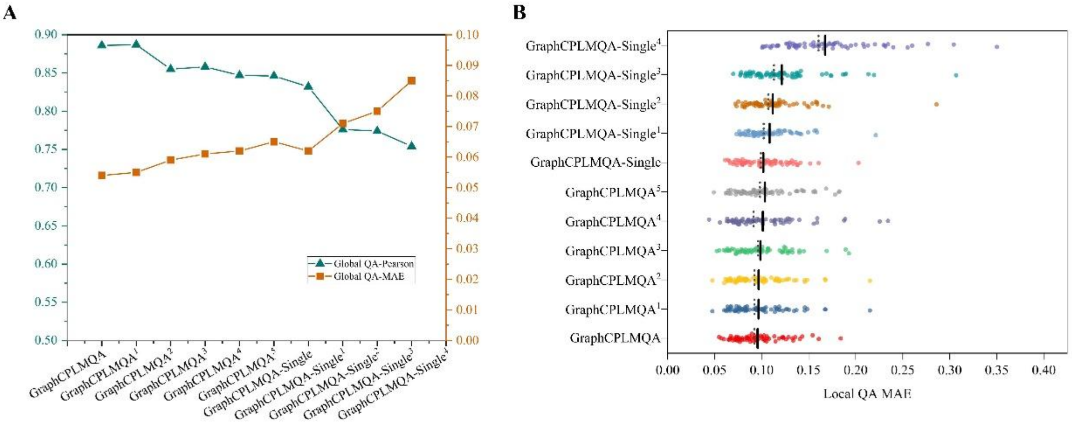

Supplementary Figure S16 Ablation studies on CASP14

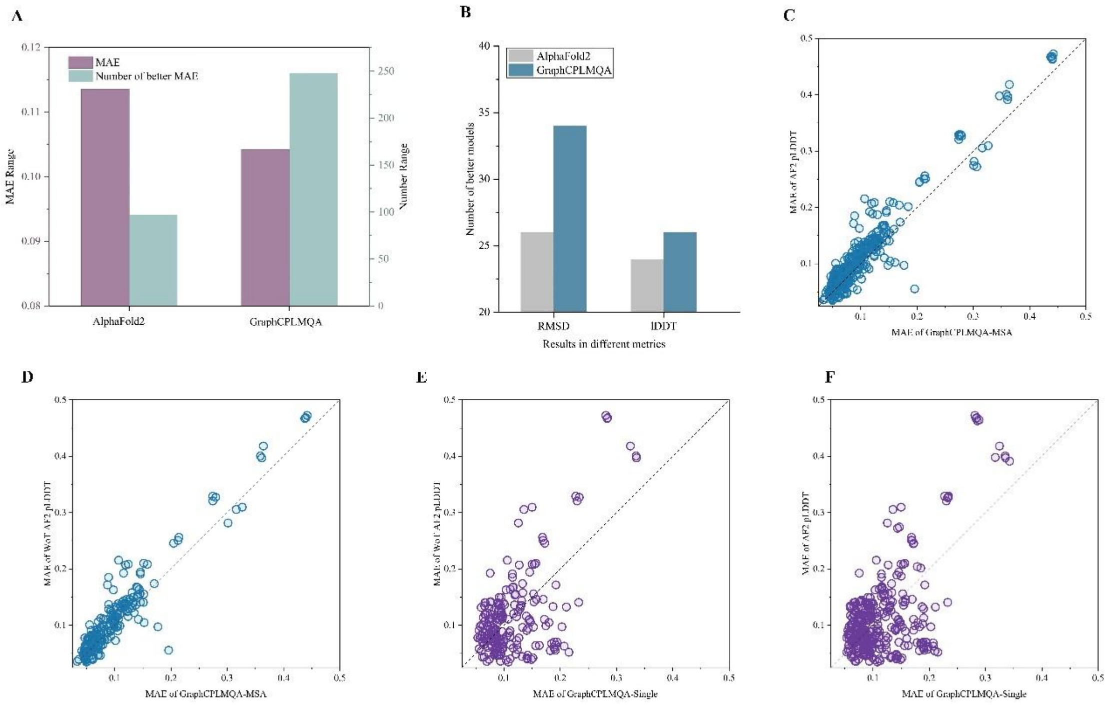

Supplementary Figure S17 Results of GraphCPLMQA and GraphCPLMQA-Single on the AlphaFold Dataset

Supplementary tables

Supplementary Table S1 ESM language model

Type	model	input	Out(dim)	Layer	Dataset
ESM-2	esm2_t33_ 650M_UR50D	single sequence	1280	33	UR50
ESM-MSA-1b [1]	esm_msa1b_ t12_100M_UR50S	MSA	768,144 (attention)	12	UR50
ESM-IF1[2]	esm_if1_gvp4_ t16_142M_UR50	backbone atomic coordinates	512	20	CATH + UR50
ESM-1v	esm1v_t33_ 650M_UR90S_1	single sequence	1280	33	UR90
ESM-1b	esm1b_t33_ 650M_UR50S	single sequence	1280	33	UR50

Note: UR is UniRef database, CATH is a classification of protein structures downloaded from the Protein Data Bank. The ESM-IF1 inverse folding model is built for predicting protein sequences from their backbone atom coordinates.

Supplementary Table S2 Feature of GraphCPLMQA

Feature	dim	Feature	dim	Feature	dim
MSA embedding	(B, L, 768), (B, L, L 144)	Relative position encoding	(5, L, K, 16)	Local Vectors and Quads	(5, L, 3)
Sequence embedding	(B, L, 1280)	Dihedral and planar angles	(5, L, 3)	Gaussian distance	(5, L, K, 15),
Structure embedding	(B, L, 512)	Voxelization and distance map	(B, L, 640), (B, L, L, 4)	Rosetta energy	(B, L, L, 7), (B, L, 7)
Triangular location and residue-level contact order	(B, L, 12), (B, L, 1), (B, L, L, 1)	Meiler, Blosum62 and DSSP	(B, L, 8), (B, L, 24), (B, L, 4)	Properties of proteins	(B, L, 27), (B, L, L, 22)

Note: In the graph coupled network, B is the training batch size (1); K is the number of neighbor nodes; L is length of protein sequence; On residues, 5 represents the five atoms C, N, O, C_α C_β.

Supplementary Table S3 Parameters of Graph Coupled Network

Parameters	Description	Value
GT-d_{n}	Graph Tansformer node dimension	64
GT-d_{e}	Graph Tansformer edge dimension	64
GT-k	Graph Tansformer attention head number	4
GT-d_{head}	Graph Tansformer attention head dimension	32
GT-d_{depth}	Graph Tansformer layer number	4
Tri-in-d_{n}	In-Triangular multiplication node dimension	64
Tri-in-d_{e}	In-Triangular multiplication edge dimension	64
Tri-in-d_{hid}	In-Triangular multiplication hidden dimension	128
Tri-out-d_{n}	Out-Triangular multiplication node dimension	64
Tri-out-d_{e}	Out-Triangular multiplication node dimension	64
Tri-out-d_{hid}	Out-Triangular multiplication node dimension	128
Seq-block	Sequence embedding block	3
IPA- d_{n}	IPA node dimension	128
IPA- d_{depth}	IPA layer number	4
IPA- k	IPA head number	8
IPA-block	IPA block	3
EGNN-globalattention- d_{n}	EGNN global attention	64
EGNN-d_{n}	EGNN node dimension	64
$\mathrm{Conv_T}$-er	Transformer-based convolution dilation rate	1,2,4,8,16
$\mathrm{Conv_T}$-IB	Inverted bottleneck	128-256-128
$\mathrm{Conv_T}$-d_{e}	Transformer-based convolution dimension	128
$\mathrm{Conv_T}$-s	Transformer-based convolution kernel size	1-3-1
$\mathrm{Conv_T}$-Main-block	Transformer-based main convolution block	3
$\mathrm{Conv_T}$-Error-block	Transformer-based error convolution block	3
$\mathrm{Conv_T}$-Threshold-block	Transformer-based threshold convolution block	3

Supplementary Table S4 70 targets protein of CASP13

T0991	T0981	T0957s2	T0988	T1017s1	T0958	T0957s1
T0984	T1019s2	T1004	T0970	T0977	T0978	T0974s2
T0967	T0955	T0961	T0998	T1010	T0968s1	T1021s2
T0974s1	T0971	T1000	T0953s2	T0979	T0987	T0954
T0983	T0986s2	T0951	T0963	T0966	T1020	T1015s1
T0982	T1008	T0989	T1014	T1016	T0985	T0990
T0976	T1018	T1015s2	T0949	T1019s1	T0973	T0962
T0980s1	T0992	T0950	T0993s1	T0986s1	T1013	T1002
T1011	T0969	T0964	T1017s2	T0997	T0980s2	T0960
T1009	T0953s1	T0975	T1021s1	T0965	T0968s2	T0959

Supplementary Table S5 69 and 34 targets protein of CASP14 and CASP15

CASP14

T1060s2	T1028	T1090	T1082	T1045s1	T1054	T1064
T1046s1	T1038	T1035	T1024	T1053	T1056	T1076
T1096	T1048	T1067	T1037	T1041	T1070	T1039
T1092	T1058	T1065s2	T1025	T1055	T1047s2	T1099
T1026	T1029	T1050	T1101	T1100	T1060s3	T1095
T1040	T1068	T1046s2	T1065s1	T1031	T1072s1	T1057
T1027	T1078	T1091	T1061	T1049	T1045s2	T1032
T1084	T1089	T1052	T1079	T1042	T1087	T1074
T1093	T1088	T1036s1	T1047s1	T1083	T1098	T1080
T1043	T1094	T1073	T1034	T1033	T1030	

CASP15

H1106	H1157	T1109	T1161
H1129	H1166	T1110	T1170
H1134	H1167	T1113	T1173
H1135	H1168	T1121	T1174
H1140	H1171	T1123	T1178
H1141	H1172	T1124	T1179
H1142		T1127	T1181
H1143		T1132	T1187
H1144		T1153	
H1151		T1160	

Supplementary Table S6 127 targets protein of CAMEO (5.20~8.13)

7Y39_B	7ZMQ_K	7W3R_A	7R20_B	7QRE_D	7OUU_B	7F4L_D	7MNV_B
7X77_A	8DI1_A	7TZV_A	7VTY_A	7VU4_A	7RT7_D	7U5O_C	7VMT_B
7DRI_A	7W7A_G	7VKK_A	7RE5_B	7R9B_A	7OU3_A	7VRC_C	7F84_A
7XKG_A	7P4L_B	7ESG_A	7F4S_D	7V2S_B	7N8U_A	8CTR_A	7EZN_A
8DYS_A	7PCR_A	7R24_A	7FAU_B	7SL5_G	7TE3_A	7SBD_H	7QVB_B
7BB8_A	7QQA_A	7XQ5_B	7P23_A	7MO1_B	7SFN_B	7S69_A	7VRS_C
7U37_A	7T9X_A	7EUS_B	7RGW_A	7RFQ_A	7T2S_A	7RDN_A	8AG9_A
7UM4_A	7SAF_B	8CWU_B	7EZB_A	7N6H_A	7F5G_B	8CU5_A	7Y6C_A
7TDR_A	7VRS_A	7VQW_A	7Y6B_A	7ZC8_A	7MNY_D	7TVC_B	7EXX_A
7MNI_C	7F15_B	7P22_A	7ZMR_K	7Y9B_A	7PLN_A	7ZMN_K	7QII_B
7P4A_A	7W6B_A	8DTE_A	7VQ6_A	7X2E_A	7FAX_A	7FCC_A	7W16_A
7UPV_A	7PCV_A	7R0R_A	7T7I_D	7PGF_D	8CSO_C	7F5K_A	7R6R_A
7ZNX_A	8DGG_A	8D1X_D	7UW7_A	7FIA_A	7TEC_A	7ZMP_K	7PB9_A
7R1K_A	7MO3_B	7OHZ_B	7TXC_E	7XH0_A	7TVY_B	7PCS_B	7XQV_B
7VF3_B	7F08_N	7VRB_A	7N7G_A	7P20_A	7QWT_A	7PGI_D	7W89_A
7N45_A	7X8C_B	7ZRO_A	7OPT_A	7XIF_D	7FAV_A	7OIO_A	

Supplementary Table S7 Performance of the method on homologous and heterologous complexes

Methods	Homo-Pearson	Heter-Pearson	Homo-MAE	Heter-MAE
**GraphCPLMA-single**	**0.655**	**0.652**	**0.144**	**0.143**
GuijunLab-RocketX	0.600	0.586	0.169	0.150
APOLLO	0.242	0.147	0.251	0.269
FoldEver	0.354	0.247	0.185	0.219
LAW	0.100	0.289	0.309	0.323
MASS	0.142	0.196	0.438	0.479
ModFOLDdockR	0.464	0.366	0.191	0.148
ModFOLDdockS	0.394	0.377	0.177	0.169
ModFOLDdock	0.265	0.209	0.286	0.191
Venclovas	0.314	0.225	0.302	0.341

Supplementary Table S8 Comparison of GraphCPLMQA with other methods on 9390 models of CASP13

**Methods**	**Local QA**					**Global QA**				
	**Pearson**	**Kendall**	**AUC**	**MSE**	**MAE**	**Pearson**	**Kendall**	**AUC**	**MAE**	**Top1 loss**
**GraphCPLMQA**	**0.860**	**0.681**	**0.930**	**0.012**	**0.079**	**0.927**	**0.777**	**0.962**	**0.044**	**0.028**
**GraphCPLMQA-Single**	**0.837**	**0.648**	**0.932**	**0.014**	**0.087**	**0.893**	**0.734**	**0.949**	**0.050**	**0.047**
DeepAcc-MSA	0.831	0.645	0.914	0.016	0.091	0.902	0.743	0.949	0.052	0.033
QMEANDisCo	0.813	0.609	0.926	0.017	0.096	0.897	0.710	0.958	0.057	0.061
DeepUMQA	0.748	0.546	0.899	0.022	0.112	0.830	0.621	0.933	0.074	0.065
ModFOLD7	0.736	0.552	0.882	0.045	0.168	0.826	0.653	0.893	0.123	0.081
DeepAcc	0.735	0.533	0.886	0.030	0.135	0.797	0.589	0.909	0.103	0.058
VoroMQA-B	0.577	0.424	0.810	0.101	0.266	0.643	0.507	0.847	0.217	0.070

Note: Results obtained by calculating all residues for all models (in the same way as the official CAMEO calculation). Pearson and Kendall represent the trend between predicted quality and true quality. AUC (Area under curve) is the area enclosed by the coordinate axis under the ROC curve. MSE and MAE reflect the difference between the predicted value and the true value. Top1loss is an indicator for selecting a model, reflecting the error between the predicted best structure and the real best structure in candidate models.

Supplementary Table S9 Comparison of GraphCPLMQA with other methods on 9645 models of CASP14

**Methods**	**Local QA**					**Global QA**				
	**Pearson**	**Kendall**	**AUC**	**MSE**	**MAE**	**Pearson**	**Kendall**	**AUC**	**MAE**	**Top1 loss**
**GraphCPLMA**	**0.784**	**0.594**	**0.882**	**0.015**	**0.092**	**0.886**	**0.721**	**0.948**	**0.054**	**0.031**
DeepAcc-MSA	0.756	0.571	0.876	0.020	0.108	0.858	0.691	0.941	0.078	0.039
**GraphCPLMQA-Single**	**0.733**	**0.544**	**0.865**	**0.018**	**0.103**	**0.832**	**0.654**	**0.939**	**0.062**	**0.048**
DeepUMQA	0.701	0.511	0.851	0.021	0.112	0.795	0.600	0.917	0.073	0.042
3DCNN	0.583	0.422	0.798	0.106	0.282	0.791	0.599	0.915	0.141	0.058
ModFOLD8	0.561	0.396	0.779	0.064	0.209	0.622	0.428	0.830	0.126	0.075
Ornate	0.474	0.335	0.742	0.068	0.215	0.665	0.497	0.856	0.173	0.056

Note: Results obtained by calculating all residues for all models (in the same way as the official CAMEO calculation).

Supplementary Table S10 GraphCPLMQA ablation experiments on 9390 models of CASP13

Methods	Local QA					Global QA				
	Pearson	Kendall	AUC	MSE	MAE	Pearson	Kendall	AUC	MAE	Top1 loss
GraphCPLMA	0.860	0.681	0.930	0.012	0.079	0.927	0.777	0.962	0.044	0.028
GraphCPLMA[1]	0.831	0.648	0.919	0.014	0.087	0.906	0.748	0.951	0.049	0.047
GraphCPLMA[2]	0.823	0.633	0.924	0.015	0.088	0.908	0.748	0.964	0.049	0.048
GraphCPLMA[3]	0.817	0.629	0.915	0.015	0.093	0.900	0.740	0.959	0.054	0.050
GraphCPLMA[4]	0.808	0.626	0.910	0.016	0.092	0.899	0.736	0.963	0.053	0.043
GraphCPLMA[5]	0.807	0.616	0.905	0.016	0.091	0.899	0.736	0.953	0.048	0.047

Supplementary Table S11 GraphCPLMQA-Single ablation experiments on 9390 models of CASP13

Methods	Local QA					Global QA				
	Pearson	Kendall	AUC	MSE	MAE	Pearson	Kendall	AUC	MAE	Top1 loss
GraphCPLMA-Single	0.837	0.648	0.932	0.014	0.087	0.893	0.734	0.947	0.050	0.047
GraphCPLMA-Single[1]	0.796	0.601	0.908	0.017	0.099	0.857	0.675	0.929	0.061	0.056
GraphCPLMA-Single[2]	0.791	0.592	0.912	0.017	0.101	0.853	0.661	0.946	0.064	0.055
GraphCPLMA-Single[3]	0.747	0.551	0.879	0.025	0.118	0.817	0.620	0.911	0.079	0.053

Supplementary Table S12 GraphCPLMQA ablation experiments on 9645 models of CASP14

Methods	Local QA					Global QA				
	Pearson	Kendall	AUC	MSE	MAE	Pearson	Kendall	AUC	MAE	Top1loss
GraphCPLMA	0.784	0.594	0.882	0.015	0.092	0.886	0.721	0.948	0.054	0.031
GraphCPLMA[1]	0.772	0.585	0.880	0.016	0.096	0.887	0.722	0.947	0.055	0.025
GraphCPLMA[2]	0.750	0.562	0.865	0.017	0.098	0.855	0.683	0.928	0.059	0.035
GraphCPLMA[3]	0.748	0.558	0.867	0.017	0.099	0.858	0.692	0.929	0.061	0.025
GraphCPLMA[4]	0.726	0.539	0.855	0.020	0.105	0.847	0.681	0.933	0.062	0.043
GraphCPLMA[5]	0.725	0.536	0.852	0.019	0.106	0.846	0.666	0.919	0.065	0.042

Supplementary Table S13 GraphCPLMQA-Single ablation experiments on 9645 models of CASP14

Methods	Local QA					Global QA				
	Pearson	Kendall	AUC	MSE	MAE	Pearson	Kendall	AUC	MAE	Top1loss
GraphCPLMA-Single	0.733	0.544	0.865	0.018	0.103	0.832	0.654	0.939	0.062	0.048
GraphCPLMA-Single[1]	0.693	0.506	0.845	0.020	0.110	0.776	0.586	0.906	0.071	0.048
GraphCPLMA-Single[2]	0.675	0.492	0.835	0.023	0.114	0.774	0.591	0.902	0.075	0.056
GraphCPLMA-Single[3]	0.656	0.474	0.823	0.028	0.130	0.754	0.567	0.903	0.085	0.050
GraphCPLMA-Single[4]	0.607	0.429	0.799	0.042	0.162	0.695	0.510	0.866	0.154	0.078

Supplementary text

Supplementary Text S1 Position encoding

In protein graph, each residue i finds the closest K residues in European space and records its index. Secondly, the index of the neighboring residue reduces the indexes of the residual i to obtain the relative neighboring residual index. Then, these relative indexes are converted into the node features of the residue through the original positional encoding [1] formula:

$$PE(pos, 2i) = \sin\left(\frac{pos}{10000^{2i/d}}\right)$$

$$PE(pos, 2i+1) = \cos\left(\frac{pos}{10000^{2i/d}}\right)$$

where *pos* is relative index; i represents the encoding dimension; d is total positional encoding dimension.

[1] Vaswani, Ashish, et al. "Attention is all you need." Advances in neural information processing systems 30 (2017).

Supplementary Text S2 Voxelization

In Euclidean space, each Ca-centered residue looks for heavy atoms within 14 Å. The global position within the 14 Å range is converted into a local coordinate system centered on Ca through the following formula:

$$v_{ji} = h_j - r_i$$

$$v_{ji}{}^{i} = lfr^{i}(v_{ji})$$

where r_i is Ca coordinate of i-th residue; h_j represents the coordinates of heavy atoms within 14A; v_{ji} is the vector whose coordinates are subtracted; lfr^i is the local coordinate system constructed by C, Ca, N within the i-th residue. At any coordinates, the relative spatial relationship of the protein structure will not change, thereby achieving rotational and translational invariance on the features. Then, the heavy atom coordinates in each local frame are discretized to the vertices of each voxel grid by trilinear interpolation which can see https://en.wikipedia.org/wiki/Trilinear_interpolation. Next, heavy atom indexes and voxelized representations are obtained from the protein structure.

Supplementary Text S3 Construction of local vectors and quadruples

we first construct a local coordinate system Γ_i^e (the construction method is the same as Γ_i). Secondly, the space vector s_{ij} between residue i and other residues j is calculated by rotating the transformation into the local coordinate system Γ_i^e to obtain the local vector s_{ij}^{Γ} . This indicates that the global spatial structure is mapped to each local space to enhance influence of protein local structure on overall topology. Moreover, in each local coordinate system Γ_i^e, we rotate the coordinate system Γ_j^e to get Γ_{ij}^e which is transformed into Q_{ij} by quadruple. The rotation transformation describes the relationship between each local spatial structure.

Supplementary Text S4 Equivariance proof process

In the network, feature f does not change its properties for orthogonal rotation matrix R and translation vector T. First, we define the model function as follows:

$$Rf^{k+1} + T, v^{k+1} = EGNN(Rf^{k} + T, v^{k})$$

In our paper, the translation process of feature *f*:

$$\left\| f_i - f_j \right\| = \left\| f_i + T - (f_j + T) \right\|$$

the rotation process of feature *f*:

$$\left\| Rf_i - Rf_j \right\| = (f_i - f_j)^T R^T R(f_i - f_j) = (f_i - f_j)^T E(f_i - f_j)$$

For the formula $f_i^{k+1} = f_i^k + \beta \sum_{i \neq j} (f_i^k - f_i^k) Wm_{ij}$ adds rotation and translation:

$$Rf_i^{k+1} + T = Rf_i^k + T + \beta \sum_{i \neq j} (Rf_i^k + T - (Rf_i^k + T)) Wm_{ij} = R(f_i^k + \beta \sum_{i \neq j} (f_i^k - f_i^k) Wm_{ij}) + T$$

Therefore, with the rotation and translation of the input feature *f*, the same rotation and translation occur at the output end.
